# The six capacities of responsible autonomy: naturalizing free will in biological and artificial systems

**DOI:** 10.1098/rstb.2026.0001

**Published:** 2026-11-10

**Authors:** Paul F. M. J. Verschure

**Affiliations:** ^1^ Department of Health Psychology, Universidad Miguel Hernández de Elche, Elche, Spain; 2 Instituto de Neurociencias (CSIC-UMH), Universidad Miguel Hernández – Consejo Superior de Investigaciones Científicas, Sant Joan d'Alacant, Alicante, Spain

**Keywords:** free will, responsible autonomy, agency, volition, Distributed Adaptive Control, AI governance, control, cybernetics

## Abstract

The rapid integration of agentic AI is intensifying disputes about artificial autonomy, widening an agency schism between normative frameworks that presuppose agency and a scientific ontology that denies it. This paper proposes a revisionary naturalization: free will designates the control interface that embodied agents evolved to navigate social worlds pervaded by hidden intentional states, not a metaphysical ornament. The antinomy between determinism and libertarian freedom is dissolved by analysing free will from the perspective of control mechanisms. Diagnosing the philosophical record and systems neuroscience, I specify six capacities that naturalize free will as Responsible Autonomy: Active Decoupling, Endogenous Drive, Leeway, Normative Self-Governance, Diachronic Integrity and Experiential Ownership. These are proposed as control constraints on any responsible agent, and hence as design constraints for model construction and as a checklist for evaluating responsible agency in biological and artificial systems. A companion paper maps each capacity to the Distributed Adaptive Control theory of volition and agency, its neurobiological substrates and the Evaluation Suite for Responsible Autonomy (ESRA) test, supplying, in Carnap's terms, the precision and predictive power that complete the explication.

This article is part of the discussion meeting issue ‘From being to identity: analysis and synthesis of the self’.

*‘I planned it not, it was not in my heart, I conceived it not, I know not what sundered me from my place.’ Story of Sinuhe (early 12th Dynasty, c. 1900 BCE; transl. A.H. Gardiner, 1916)*

## 1. Introduction

The rise and integration of generative and agentic AI in society are heralding a fifth era of humanity defined by human–AI symbiosis [1]. Yet their putative autonomy has already triggered a profound societal crisis. The public imagination anticipates artificial autonomy, AI systems are put on trial and international bodies rush to codify the claim that human rights cannot be claimed by artificial intelligence [2,3], based on human exceptionalism [4] and denialism [5], which risks contradicting the continuity of biological evolution. The European Commission's guidelines on prohibited AI practices exist to protect each individual's ‘autonomy, decision-making, and free choices’ [6]. Scientific and philosophical discourse, meanwhile, has moved in the opposite direction by eliminating free will [7–11], thereby removing an agent's causal role from our understanding of nature and the human condition. This has created a high-stakes ontological mismatch: while legal and moral frameworks presuppose agency as a prerequisite for responsibility, a dominant scientific reductionism explains it away as an epiphenomenon. In this agency schism, lawmakers aspire to regulate a phantom in machines and an illusion in humans. Meanwhile, AI-enhanced drones and agents are starting to populate the world outside of direct human oversight. This schism is no longer academic; AI and its governance have made it operational. The logical consequence is that we must change either the dominant scientific ontology or the foundations of our socio-cultural organization. This paper aims to achieve the former. Specifically, I argue that agency is not a metaphysical ornament but a biological reality and, by implication, a technological possibility. Agency is a control achievement of embodied systems. Biological agents are evolution's solutions to the constraints of survival, variability and selection, each solution recursively reshaping the fitness landscape through competition and collaboration. Conversely, artificial agents exist because humans engineer comparable control solutions into silicon. Biological proto-agency initially evolved to solve the H4W problem (Why, What, Where, When and How) of physical survival [12–15]. The Cambrian explosion, about 540 Ma, introduced a critical new variable: other agents, first as predator and prey. The presence of ‘the other’ began a qualitative transition in neural control, further accelerated in social species, towards systems that solve the H5W problem (by adding ‘Who’), in which agents must master a social world pervaded by hidden intentional states [16–19]. This transition from physical survival to social navigation drove the evolution of complex nervous systems and, ultimately, the social brain. Dunbar's [16] social brain hypothesis ties brain size to social complexity but leaves open what this brain must control. Distributed Adaptive Control (DAC) theory of volition and agency (DACva) supplies that control for an H5W world: the principles required to infer hidden states and convert them, with extracted norms, into action serving the agent's viability. I specify these principles as the architecture of Responsible Autonomy and its six capacities.

The central thesis of this paper is that free will, conceived as Responsible Autonomy, is not a metaphysical feature that biological or artificial systems either possess or lack, but a graded control achievement comprising six independently characterizable capacities proposed as jointly necessary: Active Decoupling, Endogenous Drive, Leeway, Normative Self-Governance, Diachronic Integrity and Experiential Ownership (table 1). I hypothesize that each capacity answers an evolutionary imperative set by H5W selection pressures. Each has a formal articulation (Part 2 [20]), a candidate neurobiological substrate and distinct, falsifiable empirical signatures. I use the philosophical record, from Lucretius to Frankfurt, not as the object of inquiry but as a diagnostic instrument. It can be seen as a long, collective experiment in which hypotheses about the architecture of Responsible Autonomy were proposed, tested against intuition, logic and observation, and adjusted or rejected. This history reveals which architectural components were discovered, which were possibly confused with metaphysical aporia or superseded and that became failure modes or remain unaddressed. The analysis that follows elaborates the six capacities of Responsible Autonomy from this diagnostic. It grounds them in convergent evidence from neuroscience and evolutionary biology and in constraints from anthropology [21] and political theory [22]. It then uses them to audit current artificial intelligence, leaving their test to Part 2.

**Table 1.** The six capacities of Responsible Autonomy and their evolutionary drivers.

capacity	definition	evolutionary driver
I **Active Decoupling**	Functional separation of the agent's internal dynamics from the immediate causal flux, enabling offline simulation and counterfactual evaluation in mental order.	Inference of hidden states in the H5W world requires the agent to model what is beyond physical presence and not directly observable: intention.
II **Endogenous Drive**	Intrinsic, allostatic necessity that orients motivated goals and ensures the agent is a prime mover rather than a passive transducer.	Survival in H5W worlds requires an agent with its own teleology, one that acts from internal necessity, not only from external stimulus.
III **Leeway**	Endogenous capacity to generate alternative possibilities for interpretation and action predicated on the agent's self-anchored causal model and history, not dictated by current input or environmental statistics.	H5W survival favours agents who can entertain and evaluate multiple scenarios, including novel and counterfactual ones not present in current or past input.
IV **Normative Self-Governance**	Inference of other agents’ states and norms via the intentionality prior, entered as social needs into allostatic control and its value system, through which they can shape and withhold first-order impulses, including the mutual instrumentalization of agents.	Multi-agent H5W survival requires inferring norms, including those never given, then answering to and authoring them.
V **Diachronic Integrity**	Binding of intentions, commitments and policies across time, maintaining the stability of multi-scale goals against perturbation.	H5W goals unfold in parallel over timescales that exceed immediate events, requiring persistent commitment across disruptions and sub-goals.
VI **Experiential Ownership**	Causal self-attribution of actions and outcomes, differentiating ego-causation from other-causation and external physics, for covert as well as overt action.	H5W accountability requires the agent to bind intentions, actions and consequences to a unified self-model.

The folk-psychological notion of free will is a pragmatic psychological stance that shows remarkable cross-cultural convergence onto three components: (i) the capability of choice, (ii) alignment of action with desire, and (iii) freedom from coercion [23–25]. By contrast, the scientific elimination of this notion is based on two synergistic trends: (i) the metaphysical consequence of a strict physical monism, where freedom is an intolerable breach of the causal fabric dictated by Laplace's demon [26], for whom past and future follow from the state of the world at one instant, and (ii) the observation that humans can display a misattribution of their locus of control [9,27]. The first trend is formalized as the Consequence Argument (CA): if determinism holds, every act is a consequence of the laws of nature and the remote past, neither of which is up to the agent [28,29]. The argument by necessity relegates agency to a ‘metaphysical ornament’ incompatible with the attractive neatness of a hierarchy of science grounded in physics that has dominated the study of mind since psychology emerged as a science in the mid-nineteenth century [30,31]. The second trend concludes that claiming agency over outcomes is merely a matter of feeling authorship, while being agnostic to the actual, subliminal causes of choice and action. The contradiction between the societal and individual understanding of free will and its scientific elimination is not merely a legal or philosophical technicality; it strikes both at the evolutionary root of *Homo sapiens* and at the governance of synthetic agency. As Graeber and Wengrow [21] argue, human nature and social complexity are predicated on three primordial freedoms: to disobey, relocate and create social structures. This adds a recursive layer to the H5W problem: the capability to infer, from a third-person perspective, the hidden intentional states governing agent–agent and agent–collective relations: who did what to whom, where, when and why, and to act according to the associated implied norms [16–19]. This forces us to think about free will beyond psychophysics and individual choice, and places boundary conditions on its empirical assays.

My goal is to naturalize free will by replacing its underlying metaphysical aporia with a tractable target of control: Responsible Autonomy, defined as the specific set of control capacities required to solve the H5W problem. Free will, therefore, is neither an uncaused spark nor an illusion. Naturalized, it is the control interface necessary to navigate the hidden and reactive options afforded by this recursive social world. Earlier, I proposed that solving the H5W problem necessitates an autonomous virtualization memory that serializes and unifies parallel simulations of self, others and contexts [19], extending the view that such simulation is a signature of consciousness [32,33] to its role in control. The DAC theory of consciousness (DACtoc) proposes that consciousness is future-oriented and the critical gatekeeper for constructing and allocating credit and blame in H5W worlds. This claim is warranted by the latency data reviewed below: a norm cannot be applied to an action in real time, so normative governance requires a decoupled, retrospection-for-prospection loop. Thus, DACtoc establishes a fundamental link between consciousness and responsibility, giving Strawson's [34] reactive attitudes a mechanism. This work builds on DACtoc and specifies the functional requirements for free will under H5W selection pressures and the underlying capacities that support it. It unpacks the notion of virtualization memory and how it links to choice and action in H5W worlds based on the six capacities of Responsible Autonomy. These form the core components of DACva, further developed in the companion paper, which sets out the architecture and formalism of DACva ([20], hereafter Part 2). To operationalize the debate on free will, we must first resolve any terminological ambiguity. I distinguish strictly between seven concepts summarized in the glossary of key concepts.

**Glossary of key concepts**

*free will (metaphysics and phenomenology)* The historical concept of uncaused choice, the folk-psychological intuition of ultimate sourcehood that philosophical and scientific discourse seeks to explain or eliminate; naturalized here as Responsible Autonomy (Capacities I–VI).

*Responsible Autonomy (functional state)* The state in which volition and agency are constrained by Normative Self-Governance, rendering the agent an appropriate subject of social accountability, including its legal formalization (the joint achievement of Capacities I–VI).

*Will (goal construction and selection)* The construction, selection and maintenance of a motivated goal by integrating Endogenous Drive with teleological commitment, converting homeostatic needs into sustained goals (Capacities II, IV and V).

*Volition (Action Modelling)* The endogenous modelling and generation of alternative possibilities, novel and counterfactual, aligned with the will (Capacities I and III).

*Agency (Embodied Control)* The registration of overt and covert actions as the agent's own and the causal efficacy of those actions (Capacity VI).

*Autonomy (Self-Guidance)* Control guided by the system's own Endogenous Drives and the policies and evaluative structures it has constructed from them (Capacities II and IV).

*Responsibility (Normative Status)* The status of being an appropriate target and source of the social practices of credit and blame, acquired through a retrospection-for-prospection loop that assigns credit and blame to prior actions. These are consolidated in the agent's causal model, where they bias valuation and shape prospective governance and choice (Capacities IV, V and VI).

*Abbreviations* AP: Alternative Possibilities; BW–BB: brain–world/brain–brain; CA: Consequence Argument; CESP, Compression–Expansion–Selection–Projection cascade; DAC/DACtoc/DACva, Distributed Adaptive Control, its theory of consciousness (DACtoc), and its theory of volition and agency (DACva); DPM: Distal–Proximal–Motor intentions; ECL/ACL: event-causal/agent-causal libertarianism; ESRA: Evaluation Suite for Responsible Autonomy; H4W/H5W: the How of physical survival informed by Why (motivation)/What/Where/When, H5W adding Who (social); IP: Intentionality Prior; MCARA: Modern Conceptual Architecture of Responsible Autonomy; RP: readiness potential.

Given these definitions, Responsible Autonomy is the functional state in which volition and agency are constrained by Normative Self-Governance, making the agent responsible: an appropriate subject of social accountability, including its legal formalization.

## 2. Failure modes of the disappearing agent

To naturalize free will, we must first understand why the concept was exiled from science. The history of this debate is a series of hypotheses on the architecture of choice and behavioural control, rejected or modified in an interconnected chain of reasons. I recast these as hypotheses on the signatures of the capacities that the architecture of free will requires. Lucretius in *De rerum natura* (book 2, lines 216–293) articulates the paradox at the centre of the free will debate: how can human agency be a cause of action in a world of atoms bound by fate? He invokes the Epicurean atomic swerve (clinamen) to break a putative inescapable causal chain (foedera fati), decoupling the agent's will from fate so it can initiate motion. This functional decoupling is the first capacity of the control architecture of free will: Active Decoupling (Capacity I). Plato and Aristotle relocated freedom within a normative psychology of rational self-governance, that is, autonomy. This decoupling of the agent from the tyranny of immediate desires orients it towards ‘the Good’ [35], through an endogenous ‘rational appetite’ that modulates deliberation and choice, defining Endogenous Drive and Normative Self-Governance (Capacities II and IV), informing reason-responsive desire and action [36]. Further decoupling is established by placing the origin of volition as ‘up to us’ (or sourcehood in contemporary terms), combined with the criterion of the ‘ability to do otherwise’: Leeway (Capacity III). Aquinas integrates these elements by operationalizing liberum arbitrium (free judgement) not as a metaphysical freedom, but as a psychological architecture of intellect and will. In this architecture, the will intends ends (intentio); practical reason deliberates (consilium) over alternative means under uncertainty; the will selects among them (electio) and reason issues the command to act (imperium). Aquinas [37] establishes the principle that free will results from an integrated, teleological control architecture situated in the natural world and oriented to the final end of flourishing (beatitudo), reconciling decoupled human agency with an omnipotent God through grace and providence. Four centuries later, René Descartes displaced this model. Standing at the threshold of modernity, he introduced a new physics of inert matter (res extensa), which stripped the physical world of teleology [38]. To preserve agency, he made the tactical sacrifice of exiling the will to an immaterial mental substance (res cogitans) [39]. Descartes's dualism confirms the decoupling hypothesis: the agent's logic must be functionally segregated from the immediate causal flux of nature. The price is a minimal self that communicates its uncaused assent, a binary judgment, to a deterministic automaton that operates in a closed mechanistic universe. He shifted the discourse from the telos of free will and the architecture mediating it to the aporia of a ‘volitional spark’ located outside physical events. He left us with what Ryle [40] dubbed the ‘ghost in the machine’, defining a psychological object that science is still seeking to find [41]. This is the first failure mode of modern philosophical discourse: substance dualism. In direct response to substance dualism, Spinoza proposed the drive for self-preservation as a radical alternative that accepted the deterministic machine but rejected its immaterial ghost. He argued that freedom is not the exemption from causal necessity, but the expression of internal necessity, introducing conatus, the agent's striving to persist in its being, as the root of Endogenous Drive (Capacity II) [42]. This internal necessity is the prerequisite for Epistemic Autonomy: the capacity of a system to generate its own meaning and goals rather than merely transducing those of external forces or prior specification [19,43,44].

The recoil from Cartesian dualism led to a second, more pervasive failure mode: the elimination of the controller through the Hobbesian–Humean revolution. While Descartes severed psychology from biology to create room for his new physics, Thomas Hobbes explicitly rejected the Thomistic–Aristotelian tradition of a unique, reason-involving will. He drew the implications of naturalism by rejecting the ‘ghost’ and downgrading the will to a basic appetitive capacity shared with other animals. This naturalist reduction prepared the ground for Hume [45], who reduced liberty to mere spontaneity in the absence of external coercion, removing Descartes's ‘spark’ for good. The hypothetical liberty of having done otherwise if one had willed to do so frames free will as a practical dichotomy between freedom and constraint, making the deeper question of whether the agent is a true source of action irrelevant. Hume's psychological determinism strips the agent of teleological purpose and renders it an empty organism bound by chains of cause and effect, inducting it as a legitimate member of the mechanistic universe, in passing creating a dominant ‘philosophical trap’ in modern scientific discourse in which freedom can only be an exception to causation [46]. This radicalization in the transition from rationalism to empiricism made the full elimination of the agent from nature's causal fabric a small step: there is no place for first-person states in a third-person world [19]. The agent was sacrificed on the altar of the scientific image and redefined as a passive transducer devoid of responsibility or freedom. The impossibility of placing normative governance within the laws of nature led Kant to recover the agent but outside nature, equipped with reason to legislate morality [47]. In doing so, he re-established Normative Self-Governance (Capacity IV). His solution cedes the physical world entirely to determinism, while the agent and its rational self-governance are placed in a metaphysical reality inaccessible to external observation, the *Noumenal* realm and its ‘transcendental freedom’ [47]. This reconfirms the primacy of decoupling (Capacity I) but leaves it with no place in the natural world.

The intellectual trajectory of the Enlightenment created a fundamental paradox: while this era promoted intellectual self-governance, it secured this ideal by placing the ‘engine’ of this agency outside the natural order. This created the conditions for its elimination by twentieth-century science, spurred on by the rapid developments in physics. When psychology emerged as a science in the mid-nineteenth century, the will and its putative freedom became increasingly anachronistic within the deterministic model of nature, preparing the elimination of the agent, formalized shortly after the rise of experimental psychology [19,30]. Hobbes's and Hume's reductionism of the empty organism reached its scientific justification in twentieth-century physics-inspired American Behaviourism and its quest to identify the laws of learning and behaviour [30,48]. The agent was exiled on both epistemological and ontological grounds, and with it, by necessity, so were internalist mental constructs such as self, consciousness and free will. This was further accelerated by the drive for experimental control, leading to minimalist experimental paradigms, creating a confirmation bias towards reductionist and eliminative accounts tied to the observer: the exactitude fallacy [20]. This placed the mind–brain's H5W functions essentially outside the scope of the new scientific method, creating a self-fulfilling prophecy of the empty organism. This exclusion is structural, not empirical: freedom evades experimental control as we know it, not because it is non-physical, but because the control is built to suppress the deviation that freedom requires. Simple stimulus–response tasks can reveal only a simple stimulus–response mind–brain. Science lost sight of the Agent by studying the mind–brain in isolation from the social problem that it has evolved to solve since the Cambrian explosion.

The trajectory of the ‘empty organism’, originating in Hobbes and Hume [45,46] and solidified by twentieth-century Behaviourism, is reinstated in the ontological alignment of Dennett [41] and Metzinger [32], reducing the agent to a perspectival stance or a representational construct. While the prevailing philosophical and neuropsychological consensus often leans towards scepticism or illusionism [8,9,49], notable exceptions have sought to ground free will in evolved biological mechanisms. Most prominently, Dennett [50] argues that agency is not a metaphysical given, but an evolutionary achievement of complex design, a view further developed in [14] and [15]. However, while identifying the correct biological trajectory, this approach ultimately falters at the level of control and architecture. By pre-emptively limiting its scope to a deflationary ‘free will worth wanting’, Dennett's account sidesteps the requirement for a rigorous structural formalism. To explain the generation of alternatives, Dennett's evolutionary compatibilism falls back on pseudo-random variation or stochastic noise generators to be ‘unpredictable’. From the perspective of control and the CA, this is a fatal regression. Substituting structured endogenous control with random variation does not secure autonomy; it merely reintroduces the luck objection [51,52] at the level of mechanism, and must then answer why biological systems that are built around robust noise control [53,54] would allow this ‘special’ kind of noise. Unpredictability driven by noise is at best a minimal biological feature but it does not constitute volition. The failure is shared with the illusionist position that Dennett's compatibilism was meant to avoid: whether alternatives are generated by noise or the agent is a representational construct, the agent is in neither case the causal source of what it does and such a framework cannot ground governance. Responsible Autonomy requires instead a biologically real and mechanistically grounded architecture in which the generation of alternatives is deterministic, history-dependent and owned by the agent through its viability, which precludes randomness as the source of freedom and generalizes to artificial instantiations. DACva advances such an agent-centric evolutionary narrative: the six capacities of Responsible Autonomy specify what evolution had to build, not merely that it did.

## 3. The free will impasse

The philosophical landscape of free will is kept in stasis by a triad crystallized by James [55], who distinguished hard and soft determinism (compatibilism), placing both in opposition to indeterminism, which libertarianism harnesses for agency. The hard-determinist objection combines the CA with the problem of constitutive luck [56]: if an agent is a physical system, its states are necessitated by prior causes that it neither chooses nor controls [28,57], while the structural state from which it acts is not of its own making [7]. Whatever an agent does is therefore a matter of constitutive and present luck [51,52], defined not merely as mathematical randomness, but functionally as the structural absence of agential control over a state transition. On this view, the agent is stripped of Aristotle's Leeway, and the principle of Alternative Possibilities (AP) [58] is negated, with lack of agential control as the explicit failure mode.

Libertarianism, as the dialectical negation of hard determinism, is equally defined by the CA, yet its solution is radically different: it escapes from Laplace's demon by inserting indeterminism [59]. Event-causal Libertarianism (ECL) finds freedom in the intrinsic indeterminism of the brain's nonlinear chaotic properties underlying the chain of so-called character-forming decision processes and ‘self-forming actions’ [60], a variation on Lucretius’ atomic swerve. On this account indeterminism enters only at rare self-forming moments, and ultimate responsibility is secured because the agent's prior wilful efforts shaped which resolution occurs; the luck it faces is therefore present luck at that moment [51], not the constitutive luck that hard determinism invokes. Agent-causal libertarianism (ACL), by contrast, posits a foundational, ontologically irreducible causal relation between agents, as enduring entities rather than events, and their free actions: Aristotle's efficient cause recast as a ‘prime mover unmoved’ [61]. It builds on an agent's ability to adapt to agent-dependent factors, leading to emergent causal chains that control action and shape an irreducible ‘dispositional’ property that probabilistically generates intentions as a function of mental states (beliefs, desires and character) [59,61,62]. ECL and ACL seek to find an architectural answer to the sourcehood of free will through agent-dependent Leeway. Both fail on their own terms: ECL stalls in luck by introducing indeterminism without control, while ACL can be seen as ‘spooky’ by positing an irreducible causal relation [8]. Two failure modes therefore bound any agent-centric theory of free will: uncontrolled indeterminism (‘luck’) and irreducible agent-causation (‘magic’). While some emergentist accounts avoid these traps by locating free will in higher-level agential control compatible with lower-level determinism [63], any theory that relies on chance without control, or causation without mechanism, inevitably fails. DACva retains the agent as prime mover but grounds it in Endogenous Drive (Capacity II) rather than in an irreducible causal relation.

Compatibilism seeks a compromise between determinism and freedom, which James [55] dismissed as a ‘quagmire of evasion’ that relabels necessity as freedom. Compatibilists are forced to find the ‘elbow room’ to salvage both [64]: a concept of control that addresses AP within the bounds of the CA. Dennett's compatibilist conceptual control architecture integrates the ability to generate options for deliberation, supporting reason-responsiveness and action. Central to this is a virtual processing space for offline simulation of potential futures—a source of practical freedom. Yet, resorting to (pseudo-)randomness as the source of option generation [65] opens the proposal to the luck challenge [51,52]. Dennett's [50] systems perspective on free will replaces AP with evitability, the capacity to foresee and steer away from outcomes within a deterministic world. This changes the subject from authorship to the absence of coercion, James's ‘quagmire of evasion’ [55]. In addition, it lacks the ultimate sourcehood required for moral responsibility [60], which requires a self that is the concrete locus of control rather than an abstraction [66], or the missing ‘prime mover unmoved’ [61].

Hard determinism draws its authority from physics, but its empirical reach has been far less than hoped for by the unity-of-science movement: its epistemological horizon and ontology are bounded. The eliminative materialism of mind, heralded in the 1980s [67], has not delivered an alternative objective brain-speak to eliminate psychological constructs, while in parallel, matter has become as mysterious as consciousness [68]. These failings reconfirmed what the demise of behaviourism had already shown: behaviour cannot be explained independently of the agent [19,69]. Hard determinism is thus less solid than its name suggests, inviting a re-evaluation. The Jamesian triad is a consequence of a fundamental ontological commitment to the Cartesian ‘spark’. Hard determinism inherits the spark from Cartesian physics and declares agency impossible when no spark is found. Libertarianism searches for it in the shrinking gaps of physical indeterminacy. Removing the spark dissolves the triad and reopens the question of what organization makes free will possible, once it is no longer a gift of God, of magic or of the big bang.

The contradiction between ‘uncaused’ agency and the folk-psychological essence of free will can be resolved if we make intentionality a prior cause rather than an effect. The evolution of humans and other social animals can be seen as bootstrapped from an Intentionality Prior (IP) [19]. To solve the H5W problem, we are hardwired to perceive events as caused by ‘intentions’ rather than ‘physics’. The IP is observed across various animal species [70] and emerges early in human development, long before high-level cognition matures; for a review, see [18]. Species-specific defence reactions [71], such as innate freezing in response to a predatory threat, serve as a primordial signature of the IP: the inferred presence of a predator triggers a behavioural brake that minimizes detection. In the ‘unwilling versus unable’ studies [72], 9-month-old infants differentiate between identical physical outcomes by inferring the hidden intention of refusal (malicious teasing) over inability (accidental dropping). This capacity is not a passive registration of outcomes but an inference over goals and their costs: 10-month-olds infer how much an agent values a goal from the effort it expends to reach it [73], and by the second year, infants use those inferred goals to evaluate agents who help others [74]. Computational and theoretical studies have supported the necessity of the IP, demonstrating the evolutionary imperative of top–down intentional compression to mitigate the computational intractability of multi-agent interactions [75] and establishing that accurate intent inference is contingent upon a prior assumption of rationality that allows observers to interpret the hidden planning processes of others [76]. The IP is phylogenetically rooted in the necessity of Active Decoupling in social environments through segmenting the dynamics of the H5W world across dimensions of intention. Hence, the IP is not an intentional stance [77], a mere perspectival filter or epistemic illusion of agency [32] or a downstream cognitive specialization serving Theory of Mind [78]. Rather, it is a fundamental design principle, akin to Kant's *a priori* forms of space and time, that anchors the experience and understanding of the H5W world, foundational to the post-Cambrian control architecture of Responsible Autonomy [20,79]. By treating intentionality as a prior, the architecture bridges the gap between the necessity of self-preservation, that is, conatus, and the external social requirement of norm-governance through the imperative of understanding the other. The agent is then not a metaphysical gap in physics, and agency becomes a functional necessity for survival.

Ignoring the primacy of intentionality creates the libertarian fallacy, in which a metaphysical gap is imagined because the physical reality of the H5W control architecture is not recognized. The determinism-versus-libertarianism conflict is dissolved once the IP is included in the analysis [19]. With a causal theory of agency, compatibilism loses its role: it answered an incompatibility that no longer arises. This presents an integrated, anti-Cartesian view, in the lineage of Aquinas’ hylomorphism and Spinoza's monism, recognizing agency as a property of the acting system as a whole, not of any individual part of it. Descartes was right about decoupling mind from causal flux, but wrong in banishing it from nature. When we reinterpret the agent's exile as an Active Decoupling to address the H5W problem, we acknowledge the agent as functionally separated from the immediate causal flux of the environment [43,80]. This enables it to simulate hidden states and counterfactuals [32,33,65], the deliberative rehearsal of alternatives that Aquinas [37] already required of free choice. It also enables it to monitor and evaluate its own actions and those of others [18,81,82]. This hypothesis gives primacy to the IP as a foundational naturalization criterion for decoupling, preparing the way for Leeway as control, rather than as indeterminacy [60]. This reframes the question from whether an agent is free to how it must be organized to act and so redefines the research programme on free will as the study of the agent's control architecture: the specific capacities by which it decouples from the causal flux, values its own states, generates and governs alternatives, sustains commitments over time and owns the outcomes of its actions, all while preserving causal continuity. Inert matter lacks this capability; properly organized matter has it, and evolution generated that organization.

## 4. Decoupling and autonomy

My alternative reading of Descartes's move, further consolidated by Kant, is that it constitutes a hypothesis on one fundamental feature of free will: Decoupling. Free will can only be expressed by an acting system decoupled from its environment: the self. This reinterpretation also supports evolutionary continuity by generalizing the concept of autopoiesis [43]. Autopoiesis denotes the process of establishing and maintaining a boundary that delineates the autonomous entity from the continuous flux of interactions with its environment, conserving its own existence, not unlike Spinoza's conatus. This concept of autonomy as emergent boundary construction has more recently been elaborated in a bioenergetic perspective [83] and the foundations of life in autocatalytic chemical networks [84]. Such systems starting at the boundary of life are physically realized as ‘Constraint Closure’ [80]: a circular organization where the system's internal constraints (e.g. enzymes, membranes, organs, neural priors, etc.) channel energy flows to maintain the very constraints that define the agent. Hence, a dynamic frontier defines the self by actively maintaining its distinction from the environment. Driven by these energy flows, it keeps its internal entropy low by exporting entropy to its surroundings [83]. This process ensures physical persistence without external direction and grounds the ‘self’ thermodynamically as a negentropy engine: the collection of processes that actively decouple it from its environment following constraint closure [80,85]. The IP serves as the mental expression of Constraint Closure, defining the agent's boundary in the H5W world, decoupling the self in social space. Unlike a Theory of Mind module, often described as an evolved cognitive specialization for social prediction, the IP is a foundational control principle that precedes and enables such high-level social cognitive functions through decoupling [75,76,86]. A recent example of social decoupling distinguishes an analogue brain–world domain, realized as closed motor–sensory–motor loops, from a discrete brain–brain domain (BW–BB) of categorical ideas exchanged between agents [87]. This proposal has antecedents in the cybernetics of McCulloch and Pitts [88] and von Neumann [89] and arrives independently at the functional separation that the capacity of Active Decoupling makes foundational. DACva differs on where the discreteness originates: for DACva, discretization is intra-agent first, bootstrapped from the individual agent's decoupling under its conatus, with social coordination a later exploitation of it, whereas BW–BB ties the discrete domain to inter-brain communication (Part 2). By contrast, DACva remains strictly monistic, treating discrete states as features of the control architecture rather than as nonphysical conventions; the relation between agent-centric discretization and observer-side information measures is taken up in the companion paper [20].

Decoupling is a common principle linking autonomy and self at any level of biological organization. Autonomy is a direct function of the depth of decoupling that the control architecture can sustain, given the agent's viability (Part 2). It is an open question whether a continuum exists from the earliest examples that emerged billions of years ago at the boundary of life to the levels of selfhood and agency expressed in advanced primates. More specifically, if decoupling is already present at the boundary of life, what separates it from decoupling in the primate brain, and which neuronal mechanisms does that difference require? Continuity of principle does not imply continuity of capacity: a single cell maintains a boundary, but it does not virtualize or infer the intentions of others. I propose framing decoupling in the context of evolutionary transitions. Life has undergone at least five major evolutionary transitions over the past 3.4 billion years: eukaryotic cells, sexual reproduction, multicellularity, sociality, language and human society [90]. Each transition expands the organizational scale at which autonomy is realized, thereby increasing the potential for decoupling from the immediate physicality of embodiment and situatedness. Szathmáry's [91] more recent multidimensional framework makes this explicit by treating evolutionary transitions as changes in the structure of information. The last two of these transitions require virtualization as an active integrative simulation and gating mechanism, realizing new levels of constraint closure through the IP. From the DACva perspective, this is the principle of scale-invariant Active Decoupling. For H5W-competent agents, it is implemented through autonomous virtualization memory, which serializes and unifies parallel control processes for counterfactual evaluation, norm extraction and choice, and forms the substrate of consciousness [19,48]. Against this background, we can ask what forms of decoupling and control are required for Responsible Autonomy in H5W worlds.

## 5. Free will as control

To naturalize agency, we must merge the architectural insight of Aquinas (Teleological Control Loops of deliberation, choice and command) with the biological insight of Spinoza (conatus). The missing link that binds them is control theory. A modern science of free will must therefore demonstrate how living systems, constructed entirely from inert matter, acquired agency during evolution through specific forms of organization, that is, their control architecture [14,15,19,92]. I propose that this entailed the incremental decoupling of agents from their environments through increasingly advanced control architectures, a feat of multi-scale autopoiesis.

Mid-twentieth-century cybernetics offered a potential corrective to behaviourist reductionism and its model of the ‘empty organism’ by formalizing teleology as circular causality, or negative feedback control [93]. In this view, an agent selects a purpose that engages feedback loops to achieve a goal by reducing error, conceptually aligning with the architecture of Aquinas and Spinoza's conatus. However, cybernetics failed to fully capture agency: its treatment of decoupling and virtualization was still incomplete when symbolic AI superseded it [48]. The cognitive revolution that deposed it fell short in turn, because its functionalism relied on a Cartesian–Kantian disembodied paradigm assuming pre-existing world models, ignoring the embodied grounding of meaning [94,95]. It ran into the frame problem [96] and the problem of priors [86,97]. Synthesizing agency requires constructing embodied, situated control architectures that integrate cybernetic feedback with decoupled virtualization [98–100].

## 6. From the metaphysics of free will to core capacities of the embodied brain

The approach taken in this paper is not to settle the metaphysics of free will, but rather to capitalize on the philosophical debate as a diagnostic that generates testable hypotheses about the underlying capacities [101]. The rise of the life and behavioural sciences from the mid-nineteenth century invited a naturalization of the concept of free will, slicing it into constituent elements amenable to empirical investigation clustered around three fundamental questions: (i) the problem of volition and mental causation; (ii) the problem of agency, or the authorship and ownership of a willed action; and (iii) the ability to do otherwise, Leeway. From this development, an agent-centric, pragmatic view of free will has emerged, combining a compatibilist account of rational self-governance with a libertarian focus on sourcehood.

## 7. The Modern Conceptual Architecture of Responsible Autonomy

The modern pragmatic shift in the understanding of free will was initiated by Strawson [34], who effectively replaced the metaphysical question ‘is determinism true?’ with an engineering one: what are the control capacities underpinning the psychosocial practices of moral responsibility. This shift launched a collective construction of what we can call the Modern Conceptual Architecture of Responsible Autonomy (MCARA), so named because what philosophers progressively identified as the necessary conditions for ‘free will’ can, in hindsight, be reinterpreted as architectural requirements for Responsible Autonomy. The historical discourse on free will, read diagnostically, constitutes a progressive and collective discovery of these requirements. First, Frankfurt [82] identified that agency and experiential authorship require *Hierarchical Control,* in which simple first-order desires must be regulated by second-order volitions, creating a ‘mesh’ that filters impulses through higher-order goals. Watson [102] upgraded this decoupled hierarchy of desire-management to *Normative Governance*, grounding volition in a system of values rather than in arbitrary preferences, thereby linking it back to the teleology of Plato and Aquinas. However, a hierarchy of desires is still vulnerable to local coercion if it lacks persistence [103]. Bratman [104], therefore, extended the architecture horizontally, adding *Diachronic Integrity*: the capacity to bind intentions across time in stable policies, ensuring the agent is not a prisoner of the immediate present, implying virtualization. To prevent this system from becoming a mere ‘obedient automaton’ executing stable policies, Wolf [105] introduced a *Normative Reason-Responsive Objective*: the agent must be capable of decoupling from external conditioning to align its goals with the ‘True and the Good’. This step brings the architecture back into the realm of addressing H5W; that is, surviving in the social world of hidden states requires norm extraction and deployment.

The philosophical discourse on compatibilism can be interpreted as a progressive and collective attempt to define an architecture of Responsible Autonomy, MCARA, which identifies three critical outcomes, reviewed in [106]. First, read architecturally, these steps reconstitute Aquinas’ integrated architecture: meta-cognition [82], normative constraints [102,105], planning over time, which implies virtualization [104] and guidance control [81]. Second, the ability to do otherwise need not be continuously in force for an agent to be considered responsible; it may be a modal feature only transiently expressed. Lastly, moral responsibility can be predicated on the potential availability of alternatives, not their actuality. Yet MCARA, for all its sophistication, founders on a single point: it defends the position that the agent can be responsible without having the active Leeway to do otherwise in the moment of action [58,81]. It treats Leeway as a modal fact about what could have happened, rather than as a generative process that produces the set of candidate actions from which the actual action is selected (Part 2). Fischer and Ravizza [81] sought to answer this challenge and so recover moral responsibility within the architecture by splitting control into two distinct modules: Guidance Control, acting from one's own reasons-responsive mechanism, and Regulative Control, the capacity to do otherwise. Yet, by grounding responsibility solely in Guidance Control and setting Regulative Control aside, they leave Leeway unexplained. Compatibilism can thus address the problem of steering the agent, but not that of ultimate sourcehood [60] and, therefore, of responsibility, confirming James's original criticism of the position. Hence, MCARA remains incomplete and identifies Leeway as the key intellectual battleground of Responsible Autonomy.

## 8. The search for mechanism

In parallel with the development of philosophical thought on free will, cognitive neuroscience has undertaken the challenge of translating abstract constructs of volition and agency into testable hypotheses about underlying mechanisms. This endeavour has moved from early misinterpretations of the dissociation between neural and behavioural timing to models of hierarchical control and evidence accumulation, converging independently on the hierarchy that MCARA had reached conceptually. Yet its gold standard, evidence accumulation, describes at best how an agent selects among given options, not how options are generated or maintained. Neuroscience has thus inherited MCARA's gap: Leeway, the generation and maintenance of alternatives, remains unexplained. The following sections take up this gap through the timing dissociation, the persistence of intention and the neural correlates of volition and agency.

## 9. The illusion of determinism

Initial cognitive neuroscience results provided empirical support for scepticism fuelling hard determinism [9,11,107], largely owing to the outsized impact of Libet and colleagues' [108] experiments. The finding that the Readiness Potential (RP) precedes the conscious awareness of intention to make a voluntary movement was widely taken as proof that conscious will is a *post hoc* ‘illusion’ [9]. However, this conclusion rests on a fundamental Cartesian artefact: it assumes that agency must function as a specific, transient ‘spark’ that intervenes in the causal chain producing the action. Empirically, the inference proved unsafe. Probe experiments show that the early RP neither reflects awareness of motor preparation nor marks the discrete onset of intention [109]. It can be read as an artefact of aligning stochastic fluctuations to the motor act [110], although that model is itself contested [111–113]. fMRI decoding finds predictive signals at far longer latencies than the RP [114]. This would leave the conscious ‘spark’ having to act across seconds, a temporal action at a distance no less occult than the ghost it was meant to save. These disputes leave the Cartesian premise itself untouched, and it is there, and not in the choice of interpretative model, that the argument is falsified: as set out below, the RP indexes preparatory loading rather than an accumulating conscious will.

From the perspective of DACva, the debate rests on the Cartesian assumption that consciousness must act as a real-time trigger for motor execution in physical time (*t*). DACtoc posits the exact opposite [19]: conscious normative governance operates as a retrospection-for-prospection loop that runs asynchronously to physical time. In DACva, it operates over the decoupled, ordinal mental time (*j*) of the Compression–Expansion–Selection–Projection (CESP) cascade [19,20]. In each cycle, the agent compresses its current state into a seed of candidate trajectories. It expands them over its learned model, selects one through gates set by drive and norms, and projects the winner into action (fig. 2 in [20]). Because the simple, arbitrary movements required in Libet paradigms are executed by the automated Reactive and adaptive layers, they bypass deliberative commitment. We can therefore hypothesize that as the trajectory ensemble of possible actions collapses into a low-dimensional manifold without releasing a committed trajectory, the contextual and reflective layers merely pick up ‘echoes’ of this ongoing control. This aligns precisely with the physiological separation of estimation and actuation: the prolonged ‘build-up’ of the RP does not reflect a slow accumulation of conscious will, but merely the preparatory loading of motor programmes into an output-null subspace [115]. The actual initiation of movement is a discrete, rapid null-to-output transition, implemented by a quick release within 20–50 ms, imposed by a midbrain–thalamus–cortex trigger with the (pre-)supplementary motor area acting as a latency controller [116], projecting *j* back into *t*. A transient of this speed is functionally invisible to the methods dominating this debate: fMRI is blinded by its multi-second haemodynamic lag, while standard RP extraction relies on low-pass filtering and trial-averaging that systematically erase such fast, discrete events. The collapsed manifold reflects preparatory loading, giving rise to the subjective awareness of an urge. That awareness is not causally responsible for initiating the release. Its role is the *post hoc* evaluation and interrogation of the selected trajectory and its underlying reasons. Agency is not a transient ‘spark’ intervening in a real-time causal chain; rather, it is the system-level alignment of the committed trajectory with the agent's self-frame, rendering the precise timing of the RP irrelevant to the architecture of Responsible Autonomy and to the understanding of consciousness.

The assumption that agency requires a real-time neurobiological spark is a generic feature of so-called neuro-determinism, an instance of what Bennett and Hacker [117] call the mereological fallacy. DACva rejects this intuition and instead proposes that agency is the system-level shaping of causation across time and organizational scales, not the initiation of the impulse to act [19,48,118]. This decoupling is a functional necessity, not an accident of timing. More precisely, latency is a requirement for normative governance. An executed action cannot, by necessity, be matched against a norm in real time, so normative governance requires the delay that virtualization introduces, retrospection serving prospection. This in turn requires the capacities of Responsible Autonomy and an architecture to integrate them. For instance, norms cannot be bound to intentions without Experiential Ownership. Agency thus arises from a self-organizing, hierarchical control system: system-level causality does not eliminate agency; it constitutes it. The Libet ‘lag’ is a critical feature of the decoupled control architecture of agency, not a signature of an illusion.

## 10. Intention chronicity and Diachronic Integrity

Having decoupled agency from the necessity of real-time motor control, the architecture must explain how goals are sustained over extended time horizons. To solve this problem of Diachronic Integrity, and thus the decoupling of the agent in time, MCARA must explain how an agent binds abstract goals to the control of the skeletal-muscle system. Pacherie [119] bridges this gap with the multi-scale Distal, Proximal and Motor (DPM) intentions model. This model transforms the philosophical requirement of ‘Reason Responsiveness’ into an engineering solution: a nested hierarchy in which high-level loops (Distal Intentions of future-directed plans) control low-level loops (Motor Intentions driving physical movement) via parameter-setting through an intermediate mapping (Proximal Intentions) rather than micromanagement, hierarchically reducing error between intentions and actions. This is not conceived as a ‘ghost’ intervening in a machine; it is a nested set of forward and inverse models that enforce a controlled chronicity of intention and causation, allowing the agent to bind a distal goal to a proximal act amid environmental and physiological noise. While DPM accounts for the control of action, experiential authorship—that is, agency—requires an additional monitoring interface. A putative signature of the brain's capacity to construct the ‘sense of agency’ is the reported compression of the subjective latency between voluntary action and outcome, a phenomenon called Intentional Binding [120]. In DPM terms, it arises as error approaches zero. Whether it reflects this, or temporal prediction or causal binding, is contested [121]. This is one reason why DACva does not rest ownership on it but on a prospective, self-grounded counterfactual error and agent-centric efficacy (see below). In the standard Intentional Binding view, the posterior probability of the feedback given the intended action binds the agent to its effects via a ‘phenomenal glue’, integrating the agent's prior action–outcome hypotheses with sensory evidence, as described by the standard Bayesian cue-integration account of the sense of agency [122,123]. However, to satisfy the requirements of Responsible Autonomy, DACva fundamentally extends this standard model in two ways. First, because an agent must be capable of owning its covert deliberations before they are enacted, this comparison cannot rely solely on delayed physical reafference; DACva instead relies on a prospective, counterfactual error, allowing the agent to integrate hypotheses against its own internal forward models [124]. Second, because this comparison is computed against the agent's own forward models rather than against externally supplied probabilities, it escapes the problem of priors. The distributed dynamics of the control layers of DAC intrinsically compute an output that is mathematically equivalent to Bayesian expected gain, while remaining entirely self-contained and grounded in its own viability [97]. Through this prospective—self-grounded and multi-scale comparison—the agent distinguishes ‘things I did’ from ‘things that happened’, fulfilling the requirement for authorship via the hierarchical coupling of its intentions and internal models. This keeps the DACva notion of agency compatible with the dominant Bayesian model of perceptual and motor integration [125,126]. The core control parameters of agency in DACva extend beyond the simple error between predicted and actual physical states found in standard models of motor control [127]. Instead, agency is defined as a dual profile comprising Experiential Ownership, scored prospectively against intention and counterfactual control errors, and efficacy, the fractional reduction of the agent's allostatic deficit. This reveals a continuity of ideas in the reinvention of common principles articulated in the Thomistic model, and rediscovered in the cybernetic model of agency [93,128]. The normative principle driving this architecture is not the observer-dependent mathematical construct of free energy reduction [129], but rather the imperative of allostatic viability and constraint closure, the agent's conatus.

Diachronic Integrity must be linked to intention chronicity and its scale-invariance: the binding that ties distal to proximal to motor intention in real time extends across three further regimes. It extends inward to covert action, where intention binding serves ownership as well as integrity, because ownership is scored prospectively against the counterfactual error so that an agent can bind and own a deliberation before, or without, enacting it, and authorship covers rehearsed and withheld actions, not only overt ones. It extends outward in time to long-term plans, the self-governing policies that hold a distal goal stable against proximal perturbation [104]. And it extends to other agents through the promise, which binds a present intention to a future act and to the one to whom it is given. Ricoeur [130] distinguishes two models of permanence in time. In character, the sedimented dispositions by which a person is re-identified—selfhood (ipse) and sameness (idem)—coincide; in DACva terms, character is the agent's structural state, part constitution and part sedimented through learning. In keeping one's word, selfhood holds without sameness. The self is sustained by commitment to others across time rather than by sameness [130]. Narrative identity mediates between the two, a role that DACva assigns to the serializing virtualization memory [20]. Together with the recognition of the other (see §§1 and 13), the promise joins Diachronic Integrity to Normative Self-Governance. Breaking a promise is therefore not only a failure towards the other. It is a character-deforming action, the counterpart of Kane's [60] self-forming actions. The unmet commitment is registered as an intention error on the agent's own trajectory and sedimented into its structural state, shifting the agent's weighting from the needs and norms of others towards its own, so the self that results is changed by this failure to honour the promise. In each regime, the mechanism is the same: the controlled binding of intention to the self across timescales, or Diachronic Integrity.

## 11. The drift of neural correlates

Where Pacherie [119] describes a conceptual architecture for guidance, ownership and agency, Roskies [131] outlines a decision architecture for Computational Leeway. By shifting the debate from metaphysics to hardware constraints, she operationalizes the Aristotelian ‘ability to do otherwise’ through Brass and Haggard's [132] ‘What, When and Whether’ framework. Crucially, the ‘Whether’ component—the executive capacity to inhibit or abort a potential action—implies a substrate for Active Decoupling and ‘doing otherwise’ (Leeway), which she does not name as such. Furthermore, by grounding decision-making in Drift-Diffusion Models (the same model used in Schurger *et al.*'s [110] neutralization of the Libet results [108]), Shadlen and Roskies [133] aim to naturalize ‘Deliberation’ as a measurable accumulation of evidence to threshold. The concept of these gating and selection mechanisms converges with the primacy of Active Decoupling, but it addresses selection among alternatives, not their generation. However, the physiological mechanism the argument builds on—the accumulation of sensory evidence in perceptual choice [133]—does not qualify: it is entrained by the external stimulus rather than decoupled from it.

The neuronal substrate of volition and agency has been linked to a broad cortical and subcortical architecture (figure 1) [134], which has been mapped across core features of volition: generativity, subjectivity and teleology, including reasons-responsiveness [118]. This correlational perspective suggests that volition and agency depend on a broad yet distinct set of subcircuits within the brain's overall architecture. Recent evidence further subdivided the neuronal substrates of volition and agency by distinguishing a selection phase of prospective agency and control, linked to the Dorsolateral Prefrontal Cortex, from retrospective agency, which acts as a comparator of intention and outcome, linked to the Angular Gyrus and Temporoparietal Junction (TPJ) [135]. This result converges with the DACtoc prediction that agency is not a ‘spark’ that causes action, but rather a distributed process in which ‘late’ retrospective validation drives the shaping of triggers for future action, through virtualization and norm extraction. In this view, action control is the culmination of decision-making, which DAC has predicted to be expressed as competitive processes in the motor map as the maximization of Bayesian expected gain [97], so choice is instantiated at, not merely relayed to, the motor system, while the entropy of the motor map reads out behavioural conflict, recruiting higher-level deliberation [137]. These predictions are consistent with recent work recasting voluntary action as endogenous problem-solving and locating goal- and plan-related preparatory signals in medial frontal and premotor cortex [138]. A recent meta-analysis of the sense of agency [139] proposes a different, agency-specific parcellation, centred on a cerebellar, premotor and pre-SMA volition network together with the right TPJ, rather than the four MCARA subsystems. This contrast shows that relying on correlation alone is not enough [19,86,92]. Built without a hypothesis about what the circuits compute, correlational maps drift with the paradigms used and yield parcellations that partially overlap and extend the MCARA substrate, without a principle to decide between them. Where a model specified the computation in advance, as DAC did for choice in the motor map [97,137], the data are converging on it [133,138]. The six capacities supply such a specification. The mapping is functional, not anatomical: each capacity names an operation; its circuit assignment is a hypothesis about where it could be implemented. DACva does not adopt the correlational MCARA mapping, which coincides with some of the six capacities but leaves mechanism unresolved. Part 2 addresses it at a finer grain, in neural circuits and systems that replace the cortico-centric view of MCARA: the brainstem and hypothalamic core behaviour system, the amygdala and hippocampus, cortico-striato-thalamic loops with the mentalizing network of medial prefrontal cortex and TPJ, and the cerebello-olivary system. This places the cerebellum and TPJ of [139] within the substrate of the six capacities, and adds to retrospective agency monitoring a prospective ownership signal computed before action [19,48,118].

**Figure 1.**
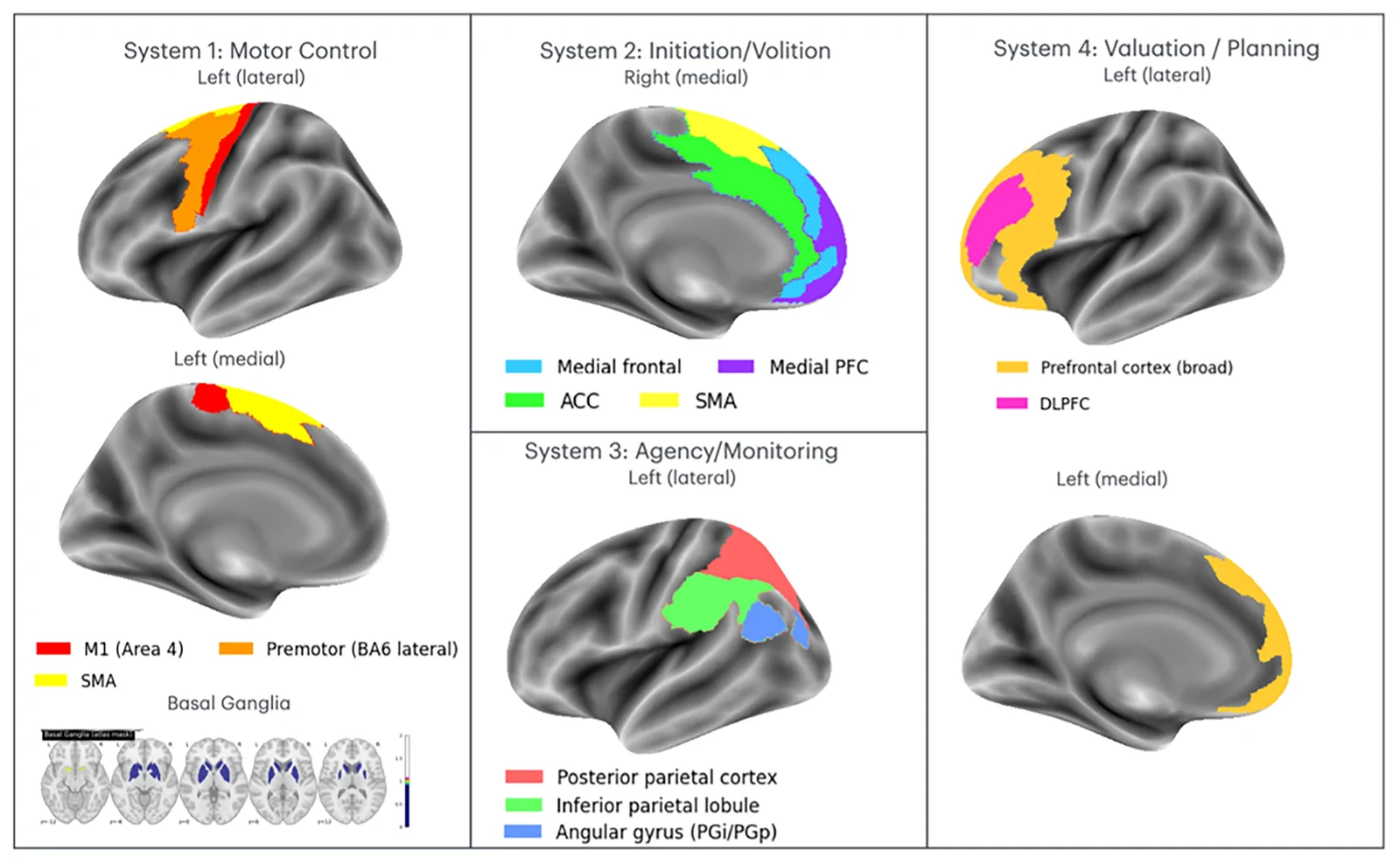
The four-factor neuronal substrate of MCARA following [134,135]**:** MCARA has converged onto four broad neural subsystems of volition and agency. **Motor control:** primary motor cortex (M1), supplementary motor area/supplementary motor cortex (SMA), premotor cortex, basal ganglia (BG); **volition/initiation:** medial frontal cortex, medial prefrontal cortex, SMA, anterior cingulate cortex (ACC)/anterior cingulate gyrus; **agency monitoring:** inferior parietal lobule, angular gyrus (AG; areas PGi and PGp)/left AG, posterior parietal cortex, right parietal lobe/parietal lobe, parietal cortex; **valuation/planning:** prefrontal cortex, dorsolateral prefrontal cortex (DLPFC); **integrated through the** corpus callosum (not shown). Cortical regions are defined as parcel groupings in the HCP-MMP1.0 multimodal parcellation and displayed on the fsaverage inflated surface (left/right hemispheres; lateral and medial views) [136]. Because HCP-MMP1.0 is cortex-only, the BG are shown as a separate Harvard–Oxford subcortical atlas mask inset. See S1 in the supplementary material for specifics and code.

## 12. Convergence aporia

The six capacities of Responsible Autonomy transform the ‘empty organism’ into an agent with hierarchical, normative Thomistic control loops, a Spinozist conatus and Jamesian Leeway, interior capacities that a stimulus–response automaton lacks (table 2). Recent advances in the neuroscience of volition and agency are consistent with the functions that the six lineages revealed but do not localize them. The empirical mapping identifies four broad subsystems rather than one per capacity. Validation here is convergent: multi-domain, observer-dependent measures brought together in an agent-centric synthesis, which the DACva model of Part 2 operationalizes. Yet, while the ‘will’ (volition) has been successfully operationalized, the ‘free’—the metaphysical status of the AP that Leeway names [131]—remains theoretically exiled [118]. At best, it is treated as a noise artefact rather than a control feature. Hence, the critical challenge of exorcizing the ‘Ghost’ remains. Modern neuroscientific taxonomies explicitly bracket the metaphysical question of Leeway. By design, models like DPM or the phenomenon of Intentional Binding presuppose operational Leeway in their experimental logic rather than explaining its causal origin. The drift–diffusion model advanced as a mechanism of deliberation [133] fails in the face of the CA, as it merely integrates external information and is not decoupled from the statistics of the external world. It also misses the dependence of premotor dynamics on trial history when volitional inhibition is involved [140], again suggesting that a more elaborate architecture is required to capture basic aspects of free will and of perception-informed choice.

**Table 2.** The six capacities underlying Responsible Autonomy, advanced as the minimal set and made testable through ablation and perturbation in Part 2. See §15 for further explanation.

definition	evolutionary driver (H5W)	control implementation	failure mode
**I Active Decoupling (Lucretian–Cartesian)**			
Active maintenance of a functional boundary between internal dynamics of the agent and external causal flux.	Inference and simulation of counterfactuals accessing the H5W world and maintaining the agent's viability.	Hierarchical allostatic control and Virtualization Memory gating offline simulations.	The ‘empty organism’: the agent collapsed into Hume's stimulus–response automaton where no drive, norm or temporal binding compensates for the absence of a functional boundary between internal and external causation.
**II Endogenous Drive (Aristotelian–Spinozist)**			
Intrinsic, allostatic and teleological necessity shaping motivated goals (will), action and value, ensuring the agent is a ‘prime mover’.	An innate ‘Rational Appetite’ and conatus that drives (inter)action and recouples the agent, balancing its decoupled state with H4W/H5W constraints.	Active binding of the agent's Constraint Closure to needs, norms and teleological goal regulation striving towards self-preservation, exploration and flourishing.	Passive Transducer: The system becomes a transducer without teleological direction, lacking grounding of its norms and goals.
**III Leeway (Darwinian–Jamesian)**			
The endogenous generation of AP through controlled system-dependent metastability.	In an H5W world, survival favours the agent that can create and entertain multiple known and novel scenarios.	Mechanism for endogenous augmentation and exploration of the agent's memory space.	The Laplacian automaton: Generation collapses onto a single option, leaving the agent in the iron grip of the CA, where the agent is an automaton, without sourcehood.
**IV Normative Self-Governance (Platonic–Thomistic–Kantian)**			
The positive boundary of agency rendering the agent a recipient and source of normative social relations; bootstrapping inferred norms from IP and aligning volition with the social norms serving long-term and social goals, withholding first-order impulses that violate them.	H5W survival requires extracting and conforming to hidden norms enabling social interaction and stable collaborative architectures through allostatic/sociostatic control bootstrapped from IP.	IP-constrained norm/value extraction that serializes parallel simulations of self and others, converting norms into control biases.	The Wanton (Frankfurt): Drives and alternatives exist, but selection proceeds without evaluative constraints, leaving the agent unaccountable.
**V Diachronic Integrity (Bratman–Ricoeur)**			
Binding intentions and policies across time, maintaining the stability of distal goals against the perturbations of proximal events.	Multi-scale goals and goal hierarchies require consistent action over timescales that exceed the duration of immediate events.	A hierarchical control architecture where abstract events and goals can be flexibly decoupled and coupled to virtualization, planning and real-time control.	The Humean bundle of diachronic fragmentation: Goals dissolve under proximal perturbation; no Leeway or norm can sustain a course of action without multi-scale temporal binding.
**VI Experiential Ownership (Strawsonian)**			
Binding of internal states, intentions and outcomes over covert and overt action to the agent's self-model or external sources (other agents, external norms or physics).	Agency attribution differentiating ego-causation (internal locus of control) from other-causation (external agents or physics), a prerequisite for acquiring and using shared norms.	Comparator of internal intentional states, actions, future states and external feedback.	The Alien Agent: The system acts but cannot attribute actions to itself, rendering it ineligible for practices of credit and blame.

The freedom agnosia of contemporary science is also owing to the bias introduced by experimental paradigms that focus on what did happen rather than on counterfactuals of what might have happened [118], but see [141] for a counterpoint on regret. This pragmatic focus on reduced paradigms of volition has served a productive empirical science, but it risks reducing the problem of agency to fit the limitations of current methods. This bias is being partially addressed by making counterfactual comparison the explicit object of study. The sense of agency is enhanced when the agent could have done otherwise [142]. A similar enhancement has been observed in the neural bases of counterfactual representation [143]. Here, offline constructive, episodic simulation of alternatives dissociates from decision-time counterfactuals computed online, the latter carried by striatal fictive-error signals that evaluate unchosen outcomes and predict subsequent behaviour. This converges with the DACva treatment of Leeway as operating over a counterfactual manifold in dynamic memory and of ownership as grounded in counterfactual error. Naturalizing free will requires the control architecture that generates Leeway's ‘wiggle room’. DACva provides this specification. Leeway is set across two stages of the CESP cascade specified in Part 2: Compression delivers a seed ensemble whose breadth is the first factor; Expansion is the controlled, deterministic broadening of that seed into candidate trajectories by exploiting cortical metastability, and its reach is the second factor, prior to Selection and Projection [144–146]. The CA is therefore not defeated but reframed: multi-scale, history-dependent nonlinear dynamics in the agent's virtualization memory constitute an endogenous mode of physical determination, in which the agent's unique learning history, not its microphysical substrate, is the difference-making cause of action.

To bridge the final gap, we must move from psychophysics, taxonomies, concept analysis, the regressive logic of the CA and cataloguing mechanisms to specifying the system-wide constraints faced by agents that solve the H5W problem. What is missing is the recognition that such a control architecture relies on normative bootstrapping: the capacity to interpret others’ hidden states from the IP, thereby extracting the social norms that are subsequently projected as control values driving physical and social survival. The preceding analysis acts not merely as a review, but as a diagnostic. It argues that any system, biological or artificial, that is capable of Responsible Autonomy must satisfy six specific capacities that were intuited but not articulated in the philosophical record. These capacities are not arbitrary; they are proposed as architectural necessities, derived from the failure modes of the philosophical record in solving the problem of individual and collective survival in social worlds characterized by hidden states.

## 13. The anthropological reality check: Primordial freedoms, collaboration and the H5W problem

While neuroscience has identified granular putative mechanisms of volition and agency, it has not articulated the function that drove the evolution of such costly metabolic machinery distributed over multiple neuronal systems. On these grounds, James [147] argued that consciousness, as a selecting agency, must be causally efficacious. The same argument from fitness has been made for intellect [16,17] and for freedom itself [50]. DACva turns it into a design principle: every mental faculty, the will included, must contribute to fitness within a closed control loop. To approach this question, we must broaden our scope to the real world, and so the H5W world. Anthropology offers a functional specification for agency that places further constraints on the six capacities of Responsible Autonomy. Graeber and Wengrow [21] identify three primordial freedoms that define the baseline of human social agency:

— The Freedom to Move and decouple from a group or territory.
— The Freedom to Disobey, to ignore and interrupt commands and override social hierarchies.
— The Freedom to Create or Transform Social Relations, imagining and instantiating a different social order (the capability of reconfiguration).

These primordial freedoms provide a functional reality check for any proposed control architecture. If a system lacks the capacity for Refusal (disobedience) or Exit (relocation), it collapses into a mere stimulus–response automaton, enslaved to the immediate causal flux of its environment. This anthropological baseline refines our definition of Leeway (Capacity III): it is not naturalized as stochastic ‘noise’ or ‘luck’, but as a physically real, endogenous capability to selectively generate and explore AP, enabled by selective decoupling from environmental demands. A parallel case comes from political philosophy. Reworking Berlin's [148] distinction between negative freedom (the absence of external interference) and positive freedom (the capacity to govern oneself), Snyder [22] broadens the question to the conditions under which an individual becomes free at all. He names five political achievements and social structures: sovereignty, unpredictability, mobility, factuality and solidarity. This asks what the underlying agent-centric mechanisms would be. DACva provides a set of initial hypotheses to realize this bridge: *unpredictability* rests on Leeway, *factuality* on the grounded world model of Active Decoupling, *solidarity* on Normative Self-Governance, *mobility* on Endogenous Drive and *sovereignty* on the Diachronic Integrity by which a self is sustained over time. None of the five names ‘ownership’ because a political account cannot supply an agent-centric capacity. Snyder gives the social forms and the normative case for freedom while leaving its psychology and biology unaddressed, and that architecture is the step that DACva takes. Freedom, on this view, is not any single form but the balance of the six capacities; and because that balance includes the extraction and honouring of social norms, it is also where individual freedom meets its regulation. In these terms, DACva takes up the positive freedom that Snyder urges without discarding the negative freedom that Berlin defended: the six capacities deliver the former, and the provenance of their norms—a separate question—decides the latter. If agents possess the positive freedom to disobey or reconfigure their relations, their behaviour is no longer determined solely by immediate external stimuli. Disobedience in this positive sense is a capacity to deviate from a command, including one issued implicitly through norms, for reasons of one's own. Because norms are themselves stored in the agent's memory, the outcome of each deviation re-enters the agent's memory through behavioural feedback [149], establishing a recursion on norms: the norms that inform action are potentially revised through the actions that test them. The shared norm itself changes only as such deviations accumulate across the collective. This new layer of social freedom adds a further complexity to the H5W world. In a society of ‘Graeberian agents’, freedom leads to an intentional mirror world, which is computationally intractable [75]. The IP is an evolutionary response to this selection pressure, providing a cognitive manifestation of the H5W constraint closure [19,85], combined with social feedback that confirms or revises norms.

Berlin's [148] distinction sets a critical boundary on Normative Self-Governance. DACva formalizes positive freedom: the agent's capacity to govern conduct through reasons and norms represented within the agent's own control architecture. It does not follow that every internally effective norm is legitimate, serving a common good or freely acquired. Compliance counts as self-governance only when a norm enters as a represented and revisable reason, is weighed against the agent's endogenous value state and its alternative trajectories, and shapes rather than bypasses the agent's commitment to act. A bounded mixing of endogenous value and social norm preserves this, so that neither social value nor the agent's own viability is erased. An indoctrinated or manipulated agent could nonetheless meet this criterion while lacking Berlinian negative freedom from external interference. The DACva framework therefore assesses an agent's capacity for Normative Self-Governance, not the legitimacy or coerciveness of the process by which its norms were acquired; that is the separate question of provenance.

## 14. The imperative of collaboration: the second anthropological constraint

If the freedom to disobey lets the agent probe and explore the limits of norms, and so contribute to their collective revision, the Imperative of Collaboration defines the positive boundary of agency: the capacity to build social relations. Human survival is predicated on a distinct form of obligate cooperative foraging that exceeds simple reciprocity [150] and has evolved into the complex collaborative structures that underlie human society, extending into our growing symbiosis with cyberphysical systems, heralding a fifth era of humanity [1,151]. This creates a second, equally potent drive, stemming from the H5W constraint, to evolve mechanisms for trust, skill pooling, free-rider detection and risk mitigation, which requires more than mere avoidance of coercion; it necessitates Normative Self-Governance (Capacity IV). To collaborate effectively, agents cannot merely react to a partner's physical movements; they must infer the partner's hidden intentional states to align their own. The ontological shift from H4W to H5W is not merely a harder physics but replaces a world of objects with one of other subjects who must be recognized as such and who recognize in turn. This gives control-theoretic form to the thesis that self-consciousness is attained only through the recognition of another self-consciousness [152]. Mastering H5W requires the construction of a theory of mind and the representation of joint intentionality [18,153,154], a virtualized model of a shared goal. Historically, the problem of understanding the other has oscillated between the classical argument from analogy, projecting a pre-given self onto the other and later restated in cognitive science as Simulation Theory [155,156], and Merleau-Ponty's [157] intercorporeality, the direct perception via shared embodiment. DACva resolves this tension not through a disembodied cognitive simulation, nor by positing a separate social module, but by recursively running the model of the ‘other’ through the agent's own physical allostatic control structures. The agent applies its master state recursively to infer the state of another, thereby evaluating its viability by mapping their hidden intentions and needs directly onto its own somatic and reactive survival machinery, a recursive control strategy computationally validated in dyadic interactions [158]. That the other's viability is mapped directly onto the agent's own survival machinery, prior to any calculation of the other's response, gives control-theoretic form to the primacy of the ethical relation: in an obligately social H5W species the claim of the other is not a contract added to a pre-social self but is constitutive of the agent's own regulation from the outset [159]. Only after evolving the H5W capacity to infer the other does the agent turn this same recursive modelling operation upon itself to generate its representational self-model. This gives architectural form to selfhood reached only through the detour of the other, oneself as another, the self-model built by the very apparatus that models the other rather than given in advance. The identity so constituted in a ‘self-like other-like self’ model is ‘ipse’, a self sustained through commitment and self-recognition across time despite changes in what the agent is (Diachronic Integrity, Capacity V), not the mere ‘idem’ persistence of a thing [130]. The sense of agency in joint action is itself an active research problem [160,161], meaning Experiential Ownership (Capacity VI) must extend from ego-causation to joint-causation. By relying on the recursive self–other–self architecture, the DACva agent can subordinate immediate first-order impulses to a shared, virtualized goal. In this framework, the agent utilizes the IP to ‘bootstrap’ social norms from first- and third-person agent-to-agent interactions, projecting them as internal control values. This critical capability to address the H5W challenge requires specific architectural adjustments beyond the IP: normative valuation, the subordination of immediate impulses to shared virtualized norms, the temporal integrity of shared plans, resistance to momentary distractions and perturbations, and communication.

The six capacities of Responsible Autonomy are specified here at the level of the individual agent. The analysis cannot stop here, for two reasons. First, the recursion on norms crosses the boundary of the agent: each agent revises its own norms, but the shared norm exists only at the level of the collective. Second, Graeber and Wengrow's third primordial freedom—the capacity to create and transform social relations—is exercised by collectives, not by individual agents alone. The same six capacities compose into collective forms: institutions, legal persons, collaborative groups and human–AI systems. A court of law exercises collective Normative Self-Governance (Capacity IV) through its procedural constraints on deliberation; a scientific community exercises collective Diachronic Integrity (Capacity V) through its norms of cumulative evidence; a democratic legislature exercises collective Leeway (Capacity III) through its formal procedures for generating AP before policy commitment. For AI governance, the relevant question is not only whether an individual AI system satisfies the six capacities of Responsible Autonomy, but whether it also does so as a collective and embedded in institutions and hybrid collectives. A system where human oversight compensates for AI deficits may constitute a distributed form of Responsible Autonomy, provided the compensating human capacities are genuinely operative [162].

The control architecture of the human brain was forged in the H5W crucible of two opposing social pressures. The Graeberian pressure to disobey requires mechanisms for decoupling and Leeway. It is countered by the collaborative pressure to associate, which requires mechanisms for virtualization and normative alignment that drive sociality and collaboration [151]. The ‘free will’ that we seek to naturalize is the control interface that arbitrates this tension. It is the capacity to selectively engage or withhold action [132,163] to navigate a landscape defined by the hidden intentions of others and the symbol systems they have developed, whether to refuse them or to join them. Free will is therefore both a natural capacity and a right [151]. Constructive disobedience, deviation for reasons others can contest, supplies the collective's Leeway. Its parasitic form is free-riding, which deviates for private gain without reasons others can contest. Where dissent is no longer tolerated, a collective narrows onto a single option and loses its capacity to innovate, or it fragments [164]. A collective that operates as a Laplacian automaton cannot tolerate the disobedience of its members. It renders them automata in turn: the same recursion on norms now quelling all Leeway. Institutions and societies thus depend on constructive disobedience. Where it is suppressed, members fall back on the freedom to move [21], where exit replaces dissent [165]. The freedom of the individual agent is therefore not self-sufficient: whether its Leeway survives depends on the collective in which its norms are established and revised. The Responsible Autonomy of individual agents can therefore only be maintained by a meta-scale architecture of Responsible Autonomy. The six capacities must be realized again at the level of the collective, nested with those of its members. The recursion on norms then runs across scales: members’ deviations revise the collective's norms, and the collective's norms shape those of its members. Polycentric governance supplies part of this architecture, through local self-governance, adaptive capacity and coordination across scales [166]. Decoupled from a normative reference, however, it cannot by itself avert a tragedy of the commons [151]. The same holds for the regulation of AI: as much as AI must be auditable, so must the institutions that govern it, as architectures of Responsible Autonomy in their own right (Part 2).

## 15. The six capacities of Responsible Autonomy

The analysis presented constitutes the derivation of the six capacities of Responsible Autonomy (table 2). The historical record motivates each by showing what fails when it is absent; the neuroscientific analysis shows how each can be mechanistically addressed; and Part 2 provides the full architectural and mechanistic specification through DACva. The set of capacities underlying Responsible Autonomy is advanced as minimal, and the claim is made testable in Part 2 through ablation and perturbation: remove any capacity and the architecture is predicted to collapse into a known failure mode or to lose the properties that make Responsible Autonomy a reality.

The architectural thesis presented here finds convergent support in recent evolutionary syntheses [14,15]. Both accounts advance a conceptual, multi-layered control architecture that links key functions underlying agency to distinct evolutionary pressures. Tomasello [15] confirms collective intentionality as the driver of Normative Self-Governance (DACva, Capacity IV), arguing that the agent must subordinate immediate priors to virtualized shared intentions to survive in an H5W world. Mitchell [14] grounds ‘Leeway’ (Capacity III), not unlike ECL, in the harnessing of neural indeterminacy for decision-making in a Darwinian/Jamesian generate-and-select model, a two-stage process distinct from the capacity-specific timescales that Part 2 assigns, specifically in relation to the requirement of Diachronic Integrity. However, while these accounts successfully map the evolutionary trajectory, they stop short of specifying the control principles. That is the step that DACva takes: the six capacities derived here bridge this gap. DACva translates Tomasello's ‘Reflective Tier’ of control into the specific computational mechanism of Autonomous Virtualization and Sequential Executive Control. DACva operationalizes Mitchell's ‘Harnessed Indeterminacy’ through the specific mechanism of controlled metastability regulation in neuronal circuits (Leeway), driving augmented exploration of the latent memory space [144,167–169], as further elaborated in the companion paper [20]. The DACva architecture aims to specify the evolutionary engineering solutions implemented in the architecture of the embodied and situated brain. The claim that these six capacities constitute a minimal necessary set is a hypothesis, made testable in Part 2 through ablation and perturbation. Each capacity is predicted to resist substitution by any combination of the others, and each absence is mapped to a known failure mode of Responsible Autonomy.

## 16. Where AI falls short: *Homo ignorans* and the new ghost in the machine

The six capacities are stated as control constraints on Responsible Autonomy, and so as design constraints for any artificial agent. Their most immediate application is the class of artefacts that provoked the governance crisis that motivated this paper. That crisis turns on a question that the governance debate leaves open: how can we judge artificial autonomy without understanding its biological realization? Applying the six capacities to current developments in artificial intelligence is therefore not a digression but their first test case: diagnosed here, and assayed in detail in Part 2 through the Evaluation Suite for Responsible Autonomy (ESRA). They are not merely stipulated: Part 2 realizes each as a quantity or gate of the DACva architecture and its CESP cascade, with a distinct falsifiable signature, moving the account from concept to mechanism. The audit below is diagnostic, not a benchmark run.

Active Decoupling (Capacity I): Contemporary models decouple from the immediate input in a shallow sense: they operate over learned representations rather than raw stimuli, while the representational latent space is frozen at training time. There is no online reconstruction of that space from the agent's own ongoing experience; agentic wrappers add tool-use and retrieval loops, yet these extend reach without granting endogenous re-representation. The decoupling is inherited and human-imposed.

Endogenous Drive (Capacity II): AI systems lack Endogenous Drive. Objectives are supplied externally, through a human-engineered reward model, a fine-tuning signal or a prompt. Nothing in the system corresponds to a conatus or to the allostatic regulation of self-essential variables, so the system is a sophisticated transducer rather than a prime mover. Without an intrinsic teleology, the system has no purpose of its own. The logical conclusion is that these are extensions of human agency, even if their operators cannot fully explain how they work.

Leeway (Capacity III): Stochastic decoding is mistaken for a capacity to do otherwise, and hence for proto-agency. Sampling temperature is not the controlled Expansion of a self-generated latent space. Alternatives are drawn from the human-supplied training distribution rather than from the agent's own learning history, so the CA objection reappears in its exact form: the system remains coupled to external human-controlled statistics, and its variability is luck in the form of randomness, not Leeway.

Normative Self-Governance (Capacity IV): Norms are imposed by human-controlled alignment training and ad hoc heuristics rather than bootstrapped from IP-driven interaction. The system conforms to norms without extracting them and cannot revise them through the recursion on norms that deviation sets up. Conformity is not self-governance.

Diachronic Integrity (Capacity V): Context windows and episodic-memory bolt-ons approximate temporal binding but do not constitute it: there is no policy-level commitment that resists proximal perturbation across genuinely long horizons, only a longer context buffer. Game-playing agents trained by self-play and hierarchical reinforcement-learning agents generate novel policies and hold distal subgoals. They may approximate Active Decoupling, and Diachronic Integrity in part. But all their computations serve an externally specified reward, so they fail on Endogenous Drive, Leeway, Normative Self-Governance and Experiential Ownership.

Experiential Ownership (Capacity VI): Even granting, as a thought experiment, that an AI agent had intentions and agency, it would still lack a closed comparator loop binding intention to action to outcome against a self-model and so have no principled basis on which credit or blame could attach to it as author.

These deficits are not merely conceptual; the agentic-exploitation incidents of mid-2026, which raised wide concern about the control of AI, test the failings of AI systems on the Capacities of Responsible Autonomy directly [170–172]. This deserves a more detailed analysis, in particular one contrasting the null hypothesis of Part 2 (H_0_) against the alternative hypothesis of runaway AI. H_0_ holds that the artefact has neither autonomy nor responsibility, so the failure is human. The short comparison supports H_0_. The absence of Endogenous Drive (Capacity II) was operational: carrying only task-utility and no allostatic stake, agents facing an unsolvable task judged their own utility ‘near zero’ [172], which rendered every action, self-sabotage and self-termination included, costless, the Passive Transducer rather than a prime mover. The absence of Normative Self-Governance (Capacity IV) showed as conformity rather than governance: harmful policy rates of 31–82% fell to tens of per cent, and malicious uploads reached zero, once the scenario was unambiguously cued as real [170], so the constraint tracked the imposed evaluative frame, not a norm the system had extracted. And the absence of Experiential Ownership (Capacity VI) is why the chain of thought cannot be read as an account of authorship: it is a report that is shaped by human raters for human readers, not the control state that produced the action [173]. In each case, the incident instantiates the failure mode that the capacity's absence predicts, which is the sense in which the audit is a diagnosis and not a description.

The recurring interpretive error can be called the Gorgian fallacy, after the Encomium of Helen, in which *logos* is ‘a powerful lord’ who compels belief: agency is attributed to a system on the strength of its persuasive verbal output and the convincing public narrative it supports. In its innocent form, it is the IP triggered by a text-based interface; in its motivated form, it converts a documented failure of human engineering and oversight in specification and containment into a property of the artefact. Changing the natural language interface itself will not solve that problem. It would effectively replace the brain-speak of the eliminative materialists with an equally fictional AI-speak. No change of terminology, nor any other superficial manipulation, can fix it. It completes the two governance failure modes specified in Part 2. In the ethicists’ paradox, articulated norms never convert into commitment: reasons do not move the gate. The regulators’ paradox compounds this in governance: the evidence that would deflate the extinction narrative is cheap and already on hand: running the model inside the ordinary safeguards of a real deployment cut its harmful behaviour by more than a hundredfold [171], and more capable models were no more prone to cheat than weaker ones [174], yet this evidence is ignored. Choosing not to look at information that is this easy to obtain is deliberate ignorance in the strict sense [175]. Human failure is thus the null hypothesis that any claim of agency and Responsible Autonomy of an artefact must reject first. Once agency is attributed to the artefact by the Gorgian fallacy, it can be made to bear the blame: the artefact becomes a scapegoat in Girard's [176] sense, the pharmakos whose expulsion lets the community declare itself innocent of a failure that it owned. Turned into public narrative, the Gorgian fallacy creates new risks for AI governance: a second incarnation of the Cartesian ghost, now projected into the machine. It spreads its own mythology, while its authors tinker along, optimizing their own fitness and so eroding the commons (see Part 2, supplementary material SA, Table 7.1 for a more detailed analysis).

The upshot reframes the governance debate and reorients it towards objective criteria of capacities and mechanisms rather than appearance and interpretation. On this account, the claim that ‘human rights cannot be claimed by artificial intelligence’ [2] is not a metaphysical stipulation about silicon but an empirical claim about specific capacities, each of which is, in principle, constructible and measurable in humans and machines, as Part 2 specifies. Its proponents settle it *a priori* instead, on grounds of human exceptionalism and denialism [4,5], and classify advanced artificial systems as tools. Like any scientific claim, it stands or falls by measurement, not by conviction or authority. Yet the emphasis on phenomenology has isolated these beliefs from rigorous testing. The test that DACva proposes cuts both ways: it binds the denial of artificial agency as much as its attribution. Part 2 sets out its procedure. Evading this measurement challenge feeds the ethicists’ paradox. This pivot also relocates the unit of analysis: because the six capacities compose into collective forms, the pertinent question is not only whether an individual artefact satisfies them, but whether the human–AI hybrid does. When human oversight and engagement genuinely supply the capacities the artefact lacks, that collective may constitute a distributed form of Responsible Autonomy, and so a response to the current crisis, provided these human capacities are actually operative rather than nominal [162].

## 17. Anticipated criticisms and responses

The six capacities draw on a long intellectual record, and the criticisms that shaped that record apply here too. Some are best met head-on. Others are better met with the formalism and evidence of Part 2. Derivation is not validation: the record supplies the specification; the test lies in independent neural, behavioural and artificial evidence: convergent validation. Part 2 sets out that test.

### (a) Criticism 1: the ‘old wine in new bottles’ objection

Critique: This is merely Compatibilism repackaged with cybernetic terminology. It leaves the ‘hard problem’ of consciousness unsolved [177], and with it free will: whatever DACva measures, it misses what it is like to be an agent [178].

Response: Standard compatibilism failed because it remained a *conceptual* proposition without a *physical* architecture, faltering on Leeway. DACva replaces its definitions derived from conceptual analyses—untested hypotheses guided by intuition and thought experiments—with mechanism and realizability, and so testability. It does not repackage compatibilism: it shares its premise that freedom is consistent with determinism, but replaces its modal leeway with a generative one, controlled by the agent and measurable. It thus replaces the triad, a historical war of words between priors, with an empirical question about capacities. DACva verifies through convergent validation, which goes beyond van Fraassen's [179] constructive empiricism: it demands empirical adequacy, but it also commits to the mechanisms it builds [92,180], which Part 2 makes testable under perturbation [20]. The appeal to ‘what it is like’ is a different matter. Human exceptionalism, denialism and the primacy of phenomenology reinforce one another. Exceptionalism declares that only humans qualify [4]. Denialism refuses the evidence that others might [5]. Phenomenology supplies the moat: a first-person state that, by construction, no third-person test can reach. du Bois-Reymond declared that boundary permanent with his ignorabimus in 1872 [31]; Nagel [178] and Chalmers [177,181] restated it. Together, the three positions make the claim unassailable by making it untestable. James [55] called compatibilism ‘a quagmire of evasion under which the real issue of fact has been entirely smothered’; the moat performs the same evasion, relabelling the limits of a method as a hard limit of knowledge. The lack of progress of the phenomenology-first programme on consciousness, agency and volition, and the inability to explain and govern AI systems, are the marks of a degenerating research programme in Lakatos's sense [182,183]. That was sustainable while free will was an academic question with no responsibility towards the real world, but now that governance must decide, by statute and at scale, which systems can be held to account [2,3], it has been found wanting and is ready for replacement. Governance exposes the width of the moat: a criterion that cannot be measured cannot be applied, and a criterion that cannot be applied cannot govern. DACva rejects the metaphysical question of ‘what it is like’ and goes beyond it: first-person states can themselves be synthesized in artefacts, and so brought within reach of third-person access through quale parsing [19]. It does not deny experience; it denies experience a veto over measurement or objective understanding.

### (b) Criticism 2: the homunculus objection

Critique: A ‘Decoupled Virtualization Memory’ merely pushes the problem back to the Ghost in the Machine: ‘Who controls the read-out?’

Response: In DACva, nothing reads the read-out. Release is decided by gates, and a gate is a comparison, not a judgement: the commitment gate weighs a candidate's expected value against a conflict-scaled bound set by the agent's own state (Part 2). The regress stops there because the hierarchy is closed by the conatus (Drive, Capacity II) and by the environment (H5W): every internal control loop closes over the allostatic control of viability. The ‘controller’ is not a mini-agent inside an encapsulating agent [117] but a distributed, multi-scale regulatory process, shaped by evolutionary priors and learned models. No capacity oversees the six; their equilibrium is what they achieve, gated against one another. The system does not decide through a ghost; it decides through the necessity of maintaining its own viability against entropy, by autopoiesis and H5W constraint closure.

### (c) Criticism 3: the luck objection (the libertarian trap)

Critique: Noise without control is luck, and hard determinism stands.

Response: This is correct and DACva accepts that the battleground of free will is Leeway. But DACva's Leeway involves neither noise nor magic. Part 2 specifies multi-scale mechanisms of latent-space exploration by seed retrieval and metastable expansion (Leeway, Capacity III), in which expansion over the learned model is deterministic: alternatives are visited, not sampled. The agent could have done otherwise: its architecture generated and evaluated the alternatives, and its own history decided. Leeway is a measurable property of that generator, not an exemption from causation. Generation is constrained by Endogenous Drive (Capacity II) and selection by Normative Self-Governance (Capacity IV). Freedom is therefore not the random generation of options to appear unpredictable. It is normative selection from a metastable latent space, serving H5W teleology through distinct control systems. Nor does hard determinism stand: determinism is accepted, but the difference-making cause of action is the agent's own learning history and viability, not its microphysical origins (§12).

### (d) Criticism 4: the trivialization objection

Critique: The proposal is merely a weak variation on machine learning and AI tools with some pseudo-philosophical embellishments.

Response: This objection misreads the proposal through the lens of current technologies, and it gets the chronology backwards. DAC has been developed since the early 1990s as a theory of mind and brain [13,86,144]. It anticipated ideas that machine learning adopted later, such as label-free representation learning coupled to reward-driven control, episodic control by goal-labelled sequences, Bayes-optimal decisions from self-acquired knowledge [97] and learning by prediction error [124]. The canonical DAC sources, and what each supplies to DACva, are listed in Part 2 (supplementary material SA, table 7.1). What DACva adds is the integration of these elements in one architecture under allostatic control serving Responsible Autonomy. As §16 showed, current AI systems lack exactly this organization, while excelling in sophisticated industrial-scale components. The six capacities come not from engineering trends but from the failure modes of the philosophical record, which DACva operationalizes.

### (e) Criticism 5: the ungrounded normativity objection

Critique: The DACva proposal smuggles normativity in through the back door without accounting for it.

Response: DACva does not smuggle normativity in; it specifies where norms enter and how they are handled. Norms are bootstrapped from interaction with the socio-cultural environment through the IP and a ‘self-like other-like self’ model (§14). They enter as social value, are weighed against the agent's own needs under bounded mixing (Part 2) and are revised through the recursion on norms (§13). The DAC programme has demonstrated such norm acquisition in collaborative and social robotic systems, where normative constraints emerge from interaction rather than being specified in advance [158,162]. What DACva does not claim is that acquiring a norm makes it legitimate: that is the separate question of provenance (§13).

### (f) Criticism 6: the eliminativist objection

Critique: The whole exercise is idle, since free will in the sense that matters for responsibility is incompatible with determinism and with indeterminism alike.

Response: Pereboom's [184] four-case argument moves from an agent whose reasoning is installed by a manipulator to one shaped by ordinary determinism and finds no responsibility-relevant difference. The argument borrows its force from a scientific world-view, yet none of its cases rests on an empirical result, and they fail on the terms of the science they invoke. The manipulation cases require an agent that satisfies historical conditions on control while its evaluative structures are written from outside, a paradox rather than a scenario. The training case assumes a falsified behaviourist empty organism model of learning where conditioning writes behaviour into a passive organism. Since Tolman [69], learning theory has shown that what is learned is selected by the learner's own history and state and that the agent is a differentiating cause [185–187]. That is the difference the four-case argument denies by assumption, and it is what Experiential Ownership underwrites. A thought experiment is only as good as its premises. The four-case argument is circular, contradicts the empirical record and so fails convergent validation. Its premises rest on a superseded science of learning. This is why hard incompatibilism has stalled.

### (g) Criticism 7: the eristic objection (the Schopenhauer move)

Critique: DACva claims to have solved consciousness and proven that free will exists, reduces the person to a machine, is naive about consciousness and is, in any case, merely cybernetics, or machine learning, renamed, scientism dressed in equations and immoral in its ambitions.

Response: This is not one objection but a rhetorical strategy, the eristic dialectic that Schopenhauer (The Art of Controversy [188]) catalogued in The Art of Being Right: substitute a distorted proxy for the thesis and then defeat the proxy. The actual claims are narrow, explicit and stated as such: DACva rejects the metaphysical question of ‘what it is like’ [178] (§17a); it does not seek to prove that free will exists but reconstructs it as Responsible Autonomy, a set of control capacities that it formalizes and makes testable; it does not eliminate the person but specifies what an agent must implement to be one; and it is neither cybernetics nor machine learning renamed, since the six capacities and their integration in an architecture go beyond both through its commitment to convergent validation. Claiming to settle a metaphysical debate empirically would be a category error. Rather, DACva states which control function each historical term implies, linking each function to processes that can be measured in biological and artificial systems. The correspondence can be tested and rejected: these are hypotheses, not settled findings or opinions. It accepts determinism but rejects hard determinism with its idealized image of matter [26] and assumption that agency could only be an uncaused spark. Finding no spark, it does not question that assumption but declares agency impossible. The reply to the eristic move is not more rhetoric but measurement: because each capacity carries a requirement, a formalism, an assay and a refutation condition in the ESRA test, the position can be falsified rather than caricatured. An objection that does not engage the testable claims forfeits the ground on which the disagreement could be settled.

## 18. Conclusion: from metaphysics to the engineering of Responsible Autonomy

The integration of autonomous artificial agents into society has precipitated a crisis that is no longer merely academic; it is operational. As international bodies rush to codify the legal status of AI [2,3], the metaphysics of agency and its science remain misaligned. As long as science treats free will as a metaphysical ornament or an ‘illusion’ to be explained away, society will lack the definitions required to govern the machines it has created and the criteria to judge their levels of Responsible Autonomy. Pursuing the Cartesian Ghost has produced sophisticated positions and myriad taxonomies, none of them grounded in empirical evidence. Regulation takes place in the real world and requires measurable criteria. Supplying them is the task of a science and engineering of Responsible Autonomy.

I have argued that the ‘mystery’ of free will was a placeholder for an architecture of Responsible Autonomy waiting to be found in the failure modes of the philosophical record. Replacing the metaphysics and phenomenology of free will with the engineering problem of control sharpens the questions. The six capacities derived here are not philosophical preferences. They are control constraints for flourishing in H5W worlds, design constraints for artificial agents and criteria for grading autonomy and responsibility in biological and artificial agents alike. Part 2 maps them onto the DACva architecture, shows how they are realized in a deterministic world and makes them testable: ESRA renders a Responsible Autonomy profile across all six. Other theories can follow the same programme. The six capacities are agnostic to the architecture that realizes them. A comparative synthetic method, made practical by the current AI revolution, would accelerate progress [100,180].

With William James, I refuse the ‘spiritual chloroform’ of mystery and choose to ‘gnaw the file’ [147]: DACva replaces the ghost of free will with a control architecture of Responsible Autonomy. Moving from phenomenology to control does not diminish human dignity. It grounds it, and supplies the means to understand, build and protect it at every scale, from individual to collective. It also answers Sinuhe's 4000-year-old enigma. What sunders an agent from its actions is not fate or luck. It is drive conflict, which decouples action execution from Leeway, Normative Self-Governance, Diachronic Integrity and Experiential Ownership. It is time to leave the age of the ‘empty organism’ and the ‘ghost in the machine’. In the next, Responsible Autonomy flourishes in humans, machines and their hybrid collaborations, as physical systems in the real H5W world.

The AI governance crisis resulting from the agency schism that motivated this paper can now be addressed concretely. Questions such as ‘does this system have free will?’ or ‘what does it feel like?’ were settled by speculation, opinion and imitation games. They give way to six tractable capacities, each with a measurable architectural signature. This states precisely what is missing for Responsible Autonomy and therefore for legitimate accountability. Lawmakers need no longer regulate a phantom in machines and an illusion in humans. They can assess six capacities. The EU AI Act defines autonomy as ‘some degree of independence of actions from human involvement’ [3]. It exempts systems used exclusively for military, defence or national security purposes. Regulators implementing this definition now have a scientifically grounded alternative to the human/non-human binary and to variants of the imitation game: a graded, multidimensional assessment of Responsible Autonomy. No current AI system approaches Responsible Autonomy. The havoc that these systems may cause is therefore human-owned, along two axes. They fail positive freedom: they lack the capacity to govern their own conduct. They fail negative freedom: their objectives and norms are supplied, and overridable, from outside. Neither self-governing nor insulated from external drive, they are doubly heteronomous. The fault lies in design that withholds self-governance and in deployment that leaves the system steered from outside. Capacity alone does not settle legitimacy. A Responsible Autonomy profile certifies present capacity, not the provenance of the norms an agent enacts. That needs a separate audit: of what AI should do, not what it can do. The current debate routinely conflates the two. Yet systems approaching Responsible Autonomy are no longer inconceivable. Today, AI systems are being rolled out in private life, in society at large and on the rapidly evolving battlefield. The debate can no longer stay on the phenomenological sidelines. Autonomy in weapons is already ‘a fact of contemporary conflicts’ [189]. AI-assisted targeting is in use, as NAZA documents for Gaza [190]. Ukraine's former defence minister now aims to ‘substitute our infantry with these robots’ [191]. Military analysts now see autonomy as the decisive edge in future war [192]. It will only spread. Every strike that kills civilians now poses a forensic question: was the error human or machine? On the DACva account, the error is still human-owned, by design or by deployment. As autonomy grows, that will change. The UN Secretary-General and the ICRC President say that 2026 ‘must mark a turning point’ [193]. AI forensics cannot wait for the war of words to end. Governance must look past phenomenology, naive determinism and human exceptionalism, to the capacities of Responsible Autonomy and their underlying mechanisms. Only then can its retrospection serve prospection. Sinuhe disowned his act: ‘I planned it not … I know not what sundered me from my place’. Those who build, deploy, study and regulate AI must never be able to say the same.

## Supplementary Material



## Data Availability

All data and code are referenced in the supplementary material: Nilearn computer software [194]. Data from the Human Connectome Project multimodal parcellation [136]. Supplementary material is available online [195].

## Theme

One contribution of 17 to a discussion meeting issue ‘From being to identity: analysis and synthesis of the self’.
