# Supplementary material for: The six capacities of responsible autonomy: naturalizing free will in biological and artificial systems
Source: Philos Trans R Soc Lond B Biol Sci. 2026 Nov 10;381(1962):20260001. doi: 10.1098/rstb.2026.0001 (PMC13750423; doi:10.1098/rstb.2026.0001)
Supplement: Supplementary file 1 [file rstb.2026.0001.zip › rstb.2026.0001/rstb20260001_si_001.docx]

# Supplementary Material S1

## Atlas-based definition of the neuronal substrate of MCARA of volition and agency following (Fried et al., 2017) and figure generation

To visualise the neuronal substrate of volition and agency and hypothetically MCARA, we defined four atlas-based systems as groupings in the HCP-MMP1.0 multimodal parcellation of the human cerebral cortex (180 areas per hemisphere). The parcellation was derived from multimodal MRI data from the Human Connectome Project and provides a high-resolution, surface-based cortical areal map suitable for reproducible region definitions (Glasser et al., 2016). Cortical parcel annotations were mapped to the fsaverage template surface and rendered on inflated cortical meshes (lateral and medial views for each hemisphere). ROI membership was represented as a surface “label texture” (an integer code per surface vertex) derived by unioning the vertices belonging to each included parcel generated by applying the parcel lists (Table S1) to the HCP-MMP1.0 annotations; these masks are the direct inputs used to render the overlays.

All grouping and rendering were implemented in Python using MNE-Python for reading surface/annotation data (Gramfort et al., 2013) and nilearn for surface visualisation (nilearn, n.d.)([nilearn.github.io](https://nilearn.github.io/dev/authors.html?utm_source=chatgpt.com)). Because HCP-MMP1.0 is a cortex-only atlas, subcortical nuclei are not represented. Accordingly, the basal ganglia were visualised separately (Motor control figure only) using the Harvard–Oxford subcortical structural atlas in standard space, displayed as an inset mask for anatomical reference ([fsl.fmrib.ox.ac.uk](https://fsl.fmrib.ox.ac.uk/fsl/docs/other/datasets.html?utm_source=chatgpt.com)).

**Table S1. Parcel-level definitions of MCARA systems (HCP-MMP1.0)**

| **System** | **Component** | **Hemisphere** | **HCP-MMP1.0 parcels included** |
| --- | --- | --- | --- |
| 1 Motor control | M1 | bilateral | 4 |
| 1 Motor control | SMA | bilateral | 6ma, 6mp, SCEF, SFL |
| 1 Motor control | Premotor | bilateral | 6d, 6v, 6r, 6a, 55b *(optional: FEF, PEF)* |
| 2 Initiation/volition | Medial frontal | bilateral | 8BM, 8BL, d32, p32, s32 |
| 2 Initiation/volition | Medial PFC | bilateral | 9m, 10v, 10r, 10d, a10p, p10p |
| 2 Initiation/volition | ACC | bilateral | 24dd, 24dv, 24cd, 24cv, p24, a24, p24pr, a24pr, 33pr, p32pr, a32pr |
| 2 Initiation/volition | SMA | bilateral | 6ma, 6mp, SCEF, SFL |
| 3 Agency monitoring | Posterior parietal | bilateral | IPS1, IPS2, IP0, IP1, IP2, AIP, MIP, VIP, LIPd, LIPv, 7AL, 7PC, 7A, 7PL, 7PM |
| 3 Agency monitoring | IPL | bilateral | PF, PFm, PFop, PFt, PFcm |
| 3 Agency monitoring | AG | left | PGi, PGp |
| 4 Valuation/planning | PFC (broad) | bilateral | 10v, 10r, 10d, a10p, p10p, 9m, 9a, 9p, 8Ad, 8Av, 8BL, 8BM, 8C, 44, 45, 47l, 47s, IFJa, IFJp, IFSp, IFSa, 11l, 11m, 13l, 13m |
| 4 Valuation/planning | DLPFC | bilateral | 9-46d, 46, a9-46v, p9-46v |

*Note: parcel names are given as base labels; hemisphere-specific labels correspond to L_<parcel> and R_<parcel> in the atlas annotation. Not all generated surfaces are shown in the figure.*

**Table S2 . Subcortical labels used for the BG inset in Figure 1**

| **Figure panel** | **Atlas** | **Space** | **Labels included** |
| --- | --- | --- | --- |
| Fig. 1 inset | Harvard–Oxford subcortical structural atlas | standard (MNI) | caudate, putamen, pallidum, accumbens (left/right; as available in the atlas build) |

**Data accessibility.** No new empirical data were generated for this study. The parcel lists defining each MCARA system (Table S1), the code used to generate the atlas-based surface masks and figures (Code S1), and the derived ROI masks used for visualisation are provided in the electronic supplementary material.

Python code available upon request.

# References

*Nilearn* [Computer software]. https://doi.org/https://doi.org/10.5281/zenodo.8397156

Fried, I., Haggard, P., He, B. J., & Schurger, A. (2017). Volition and Action in the Human Brain: Processes, Pathologies, and Reasons. *Journal of Neuroscience*, *37*(45), 10842–10847.

Glasser, M. F., Coalson, T. S., Robinson, E. C., Hacker, C. D., Harwell, J., Yacoub, E., Ugurbil, K., Andersson, J., Beckmann, C. F., & Jenkinson, M. (2016). A multi-modal parcellation of human cerebral cortex. *Nature*. 536, no. 7615 (2016): 171-178

Gramfort, A., Luessi, M., Larson, E., Engemann, D. A., Strohmeier, D., Brodbeck, C., Goj, R., Jas, M., Brooks, T., Parkkonen, L., & others. (2013). MEG and EEG data analysis with MNE-Python. *Frontiers in Neuroinformatics*, *7*, 267.

NOTES:

Based on the review of the **Supplementary Material S1**, the document is generally consistent with the main manuscript's "Figure 1" description and standard neuroimaging reporting practices. It provides the necessary technical definitions for the cortical parcellations (HCP-MMP1.0) and subcortical regions (Harvard-Oxford).

However, there are a few specific **formatting errors** and **functional definitions** you should review before final submission:

\### 1. Typo in References

There is a URL formatting error in the **Nilearn** reference.

*

**Current Text:** `https://doi.org/https://doi.org/10.5281/zenodo.8397156`

* **Correction:** Remove the duplicate protocol. It should read: `https://doi.org/10.5281/zenodo.8397156`

\### 2. Functional Overlap (SMA)

In **Table S1**, the **SMA** (Supplementary Motor Area) parcels (`6ma, 6mp, SCEF, SFL`) are listed in **both**:

*

**System 1 (Motor control)**

*

**System 2 (Initiation/volition)**

**Note:** While biologically accurate (SMA bridges volition and execution), if you are rendering these as distinct colors in a figure (as implied by Figure 1 in the main text), this overlap will cause "Z-fighting" (flickering) or color blending unless one layer explicitly masks the other. Ensure your Python code handles this priority (e.g., does Volition overlay Motor, or vice versa?).

\### 3. Lateralization of Agency (Angular Gyrus)

In **Table S1**, **System 3 (Agency monitoring)** restricts the **Angular Gyrus (AG)** specifically to the **left hemisphere** (`AG, left`), while other components like IPL are `bilateral`.

**Scientific Check:**

* The main manuscript text mentions "retrospective agency... linked to the Angular Gyrus (AG) and Temporoparietal Junction (TPJ)".

* **Observation:** Much of the literature (e.g., Farrer et al., 2003) links **agency mismatch detection** strongly to the **Right TPJ/AG**. The **Left AG** is often associated with *intentional binding* and *motor intention*.

* **Action:** Verify if you intend to exclude the **Right AG** from the "Agency monitoring" mask. If following Fried et al. (2017) strictly, ensure this aligns with their specific ROI definition.

\### 4. Code Availability

The statement "Python code available upon request" contradicts the earlier statement: "the code used to generate... are provided in the electronic supplementary material".

* **Recommendation:** If the code is uploaded as a file (e.g., `Code S1`), remove the phrase "available upon request" to avoid ambiguity.

\### 5. Formatting Consistency

* **Glasser et al. (2016):** The citation format `536, no. [cite_start]7615 (2016): 171-178` differs from the style of other references (e.g., `37 (45), 10842–10847` ). You should standardize this to match the journal's required citation style.
